# Probing the structural pathway of conformational polymorph nucleation by comparing a series of α,ω-alkanedicarboxylic acids

**DOI:** 10.1107/S205225252000233X

**Published:** 2020-03-26

**Authors:** Peng Shi, Shijie Xu, Yiming Ma, Weiwei Tang, Feng Zhang, Jingkang Wang, Junbo Gong

**Affiliations:** a School of Chemical Engineering and Technology, State Key Laboratory of Chemical Engineering, Tianjin University, Tianjin 300072, People’s Republic of China; b The Co-Innovation Center of Chemistry and Chemical Engineering of Tianjin, Tianjin 300072, People’s Republic of China; c Tianjin Key Laboratory of Marine Resources and Chemistry, College of Chemical Engineering and Materials Science, Tianjin University of Science and Technology, Tianjin 300457, People’s Republic of China

**Keywords:** conformational polymorphs, nucleation routes, α,ω-alkanedicarboxylic acids, self-assembly, conformational rearrangement, intermolecular interactions, polymorphism, crystallization and crystal growth

## Abstract

Herein the nucleation pathway of conformational polymorphs was revealed by studying the relationships and distinctions among a series of α,ω-alkanedicarboxylic acids [HOOC–(CH_2_)_*n*−2_–COOH, named DA*n*, where *n* = 5, 7, 9, 11, 13, 15] in the solid state and in solution. Their polymorphic outcomes, with the exception of DA5, show solvent dependence: form I with conformation I crystallizes from solvents with hydrogen-bond donating (HBD) ability, whereas form II with conformation II crystallizes preferentially from solvents with no HBD ability. In contrast, form II of DA5 does not crystallize in any of the solvents used. Quantum mechanical computation showed that there is no direct conformational link between the solvents and the resultant polymorphic outcomes. Surprisingly, solute aggregates were found in no-HBD solvents by Fourier transform infrared spectroscopy, and only monomers could be detected in HBD solvents, suggesting stronger solvation. Furthermore, it was found that all six compounds including DA5 followed the same pattern in solution. Moreover, crystal-packing efficiency calculations and stability tests stated that dimorphs of DA5 bear a greater stability difference than others. These suggest that the rearrangement from conformation II to I could not be limited by hard desolvation in HBD solvents, where form I was also obtained. In other systems, metastable II was produced in the same solvents, probably as a result of the rearrangement being limited by hard desolvation. In this work, a comparative study uncovers the proposed nucleation pathway: difficulty in desolvation has a remarkable effect on the result of rearrangement and nucleation outcome.

## Abstract 2

The relationships and distinctions among a series of diacids in polymorphic outcomes and solid/solution chemistry were utilized to reveal the conformational polymorph nucleation pathway: difficulty in desolvation has a remarkable effect on the result of rearrangement and nucleation outcome.

## 1. Introduction   

Polymorphism refers to a common phenomenon where the same substance crystallizes in different crystal structures which affects the chemical and physical crystal properties (Hollingsworth, 2002 ▸; Desiraju, 1997 ▸). Thus, the prediction and control of polymorph nucleation (Desiraju, 1997 ▸) has always been an important but challenging goal for researchers faced with an unclear nucleation mechanism (Kulkarni, Meekes & Horst, 2014 ▸; Kulkarni, Weber *et al.*, 2014 ▸; Li *et al.*, 2019 ▸; Rath *et al.*, 2007 ▸; Xu *et al.*, 2016 ▸; Zuñiga *et al.*, 2018 ▸; Myerson & Trout, 2013 ▸; Davey, 2004 ▸), and it is an important probe to investigate nucleation mechanisms and pathways in solution crystallization.

At present, the structural relevance between solute self-assemblies in solution and molecular synthons in the resultant crystals is of great interest (Parveen *et al.*, 2005 ▸; Burton *et al.*, 2010 ▸; Tang *et al.*, 2017 ▸; Zeng *et al.*, 2018 ▸) and could shed light on how nucleation proceeds. For instance, it was found that the formation of two conformational polymorphs of *N*-phenylhydroxamic acid had direct correspondence with the dominant conformers in two different solvents (Yamasaki *et al.*, 2006 ▸; Ischenko *et al.*, 2005 ▸), which suggested a simple nucleation pathway. Jansen and coworkers (Ischenko *et al.*, 2005 ▸) reported that a metastable conformational polymorph of 1,1,3,3,5,5-hexachloro-1,3,5-trigermacyclohexane could only be formed in *n*-hexane, and based on the crystal structure analysis and computer simulations, they put forward a route involving an intermediate solvate with a favorable lattice energy and close structural relationship with the metastable form. Davey and coworkers (Back *et al.*, 2012 ▸) presented a possible nucleation mechanism for ethenzamide with a high-energy conformer by combining *ab initio* predictions, solution Fourier transform infrared spectroscopy (FTIR), nuclear magnetic resonance and polymorph screening, that is, crystallization from solutions involves ethenzamide molecules rearranging on contact with the crystal surface, rather than the crystal growing from a very low concentration of the high-energy conformer.

However, there is still controversy about whether there is a correlation between the solute species in solution and solid structures. In some systems, it was found that the molecular packing styles (Desiraju, 2014 ▸) or conformation in crystal structures could be predicted directly according to the self-association modes of solute molecules or conformers in solution (Parveen *et al.*, 2005 ▸; Chadwick *et al.*, 2009 ▸; Kulkarni *et al.*, 2012 ▸; Mattei & Li, 2012 ▸). In a number of other examples, restructuring may happen because the link between solute species in solution and the solid structures is missing (Back *et al.*, 2012 ▸; Davey *et al.*, 2013 ▸; Du *et al.*, 2015 ▸). Overall, a systematic theoretical basis for the nucleation pathway is still absent since the nucleation process investigation depends heavily on the chosen system which is too fragmented. Therefore, exploring the nucleation pathway of polymorphs with a series of similar and comparable compounds as models may further advance our understanding and control of nucleation behavior based on their relationships and distinctions.

**Figure 1:**
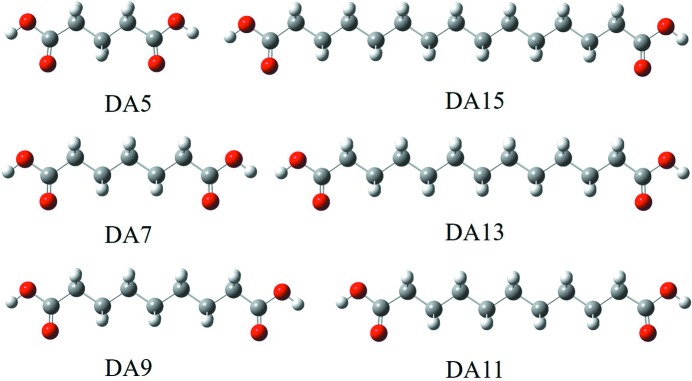
Chemical structures of six dicarboxylic acids.

In this contribution, odd-carbon α,ω-alkanedicarboxylic acids [HOOC-(CH_2_)_*n*−2_-COOH, *n* = 5, 7, 9, 11, 13, 15] were selected as research models because these diacids usually crystallize in two modifications, whereas even-carbon diacids do not follow the same pattern. For the convenience of description, herein the six diacids (Fig. 1 ▸) will be described in the style of ‘DA + the number of C atoms’. For instance, glutaric acid (*n* = 5, GA) and pentadecanedioic acid (*n* = 15, PDA) are represented by ‘DA5’ and ‘DA15’, respectively. Glutaric acid (DA5), pimelic acid (DA7), azelaic acid (DA9) and tridecanedioic acid (DA13) were reported to crystallize in different forms (Espeau *et al.*, 2013 ▸; Housty, 1968 ▸; Delarbre *et al.*, 1989 ▸; Kay & Katz, 1958 ▸) and two polymorphs of undecanedioic acid (DA11) were also prepared successfully in our recent work (Shi *et al.*, 2018 ▸). Thus, the diacids of this homologous series show some similarities and are comparable, although polymorphism of DA15 has not been reported. However, there is no research on their polymorph formation in solution in addition to our work on the mechanism of solvent-dependent conformational polymorph selectivity of DA11 (Shi *et al.*, 2018 ▸), where solvation, conformational rearrangement of solute molecules and the polymorph nucleation outcome were correlated as the nucleation pathway. Therefore, this homologous series was chosen to probe further into the nucleation pathway of conformational polymorphs. Firstly, different solvents, which possess different hydrogen-bond donating (HBD) and hydrogen-bond accepting (HBA) abilities, are employed to explore their role in the nucleation process and outcome. Secondly, we aim to explore and compare six compounds in the context of solid and solution chemistry. Finally, with the similarities and distinctions of a series of models, we can probe and further understand the nucleation pathway of conformational polymorphs.

## 2. Experimental   

### 2.1. Materials   

DA5, DA7 and DA9 (99% purity) were obtained from Aladdin Industrial Co., Ltd, China. DA11, DA13 and DA15 were supplied by Shanghai D&B Biological Science and Technology Co. Ltd, China, with a mass fraction purity >97%. Organic solvents in Table 1 ▸ were purchased from Tianjin Jiangtian Chemical Reagent Co., Ltd, China. The solvents were analytical grade with purity >99.5%. All of the materials above were used without further purification.

### 2.2. Characterization   

#### 2.2.1. Powder X-ray diffraction   

Powder X-ray diffraction (PXRD) data in this study were obtained on a Rigaku D/MAX 2500 X-ray diffractometer (Rigaku, Japan) by utilizing Cu *K*α radiation (λ = 1.54178 Å) at 100 mA and 40 kV to determine the crystalline forms of the solid samples. Data were acquired at ambient temperature (298.15 K). Samples were scanned in the 2θ range 2–40 or 2–25° with a scan speed of 8° min^−1^ in reflection mode.

#### 2.2.2. Single-crystal structure determination   

Single-crystal structure investigation was conducted on a Rigaku 007HF XtaLAB P200 diffractometer equipped with a rotating anode system by utilizing graphite-monochromated Mo *K*α radiation (λ = 0.71073 Å). *CrystalClear* (Rigaku, 2013 ▸) was used to collect data and refine the cell parameters. *SHELXS* and *SHELXL* (Sheldrick, 2014 ▸, 2015 ▸) were used for solving and refining the structure, respectively. All non-hydrogen atoms were refined anisotropically. Hydrogen atoms were assigned idealized positions and were included in structure factor calculations.

#### 2.2.3. Fourier transform infrared spectroscopy analysis   

FTIR spectra were collected for liquid samples on an FTIR spectrometer (ReactIR^TM^45, Mettler-Toledo) under ambient conditions. The wavenumber range used was 2800 to 600 cm^−1^ and the resolution was 4 cm^−1^. The background of the corresponding solvent in liquid samples was deducted when collecting spectrograms of the samples.

### 2.3. Solubility measurement   

The necessary equilibrium solubility determination was carried out in the crystallization experiment design. The solubility data of form I of the six diacids in 1,4-dioxane, DC11 and DC15 in ethanol, acetic acid and ethyl acetate were determined by gravimetric analysis. The solubilities of metastable form II diacids were determined by the dynamic method using the laser monitoring observation technique. Other necessary data relating to equilibrium solubility were obtained from previous work (Tang *et al.*, 2015 ▸; Zhang *et al.*, 2014 ▸) by the same measurement method. Details of the experimental apparatus and process of gravimetric analysis (Shi *et al.*, 2019 ▸) are given in the supporting information and the solubility data are given in Table S1.

### 2.4. Polymorph formation experiments   

The polymorphic formation of six diacids was studied in four different pure solvents (ethanol, acetic acid, ethyl acetate and 1, 4-dioxane) and in two different supersaturations (*S*): 1.3 and 2.5 (*S* = *C*/*C*_s_; *C* = actual concentration, *C*_s_ = solubility at 298.15 K) by isothermal cooling crystallization. A given mass of diacids was added to 10 g pure solvent based on the desired *S* at 298.15 K and heated to elevated temperature (338.15 K) to dissolve the solute completely. Then the heated solutions were filtered through a preheated 0.22 µm syringe filter and transferred into a glass test tube and held at 298.15 K for 30 min. The tubes were then rapidly transferred into a thermostatic water bath (model 501 A, Shanghai Laboratory Instrument Works Co., Ltd, China) with an accuracy of ±0.05 K while agitating (400 rpm) by a magnetic stirrer, rapidly resulting in solutions of *S* = 1.3 and 2.5 at 298.15 K. After the induction stage of nucleation, a solid appeared. This was then separated from the suspension as soon as possible and analyzed by PXRD to determine the crystal form. Each experiment was repeated three times.

### 2.5. Additive disturbance experiments   

Additive disturbance experiments were designed to verify the relationship between solute self-aggregation and the formation of two forms of a conformation. A given mass of additives (adipic acid and benzoic acid) and form I of DA5-11 in different molar ratios were added to 3 g pure 1,4-dioxane and heated to 323.15 K to ensure the solute dissolved completely. Then the solutions were filtered through a preheated 0.22 µm syringe filter and transferred into a jacketed vessel and held at constant elevated temperature for 30 min. The solution system was then cooled to 288.15 K at a cooling rate of 0.15 K min^−1^ controlled by a thermostat (model 501 A, Shanghai Laboratory Instrument Works Co., Ltd, China) with an accuracy of ±0.05 K while stirring (300 rpm) using a magnetic stirrer. The solid obtained, which was analyzed by PXRD to identify the form, was separated from the suspension as soon as possible after nucleation during cooling to avert the underlying polymorphic transition. Each experiment was repeated three times.

### 2.6. Stability experiments of the polymorphs of six diacids   

Both forms of DA7, DA9, DA11, DA13 and DA15 were obtained through solvent dependence. Form II of DA5 was prepared by melt quenching. Two forms of DA5–DA15 were stored in glass dishes at ambient temperature and humidity. The crystal forms were determined by PXRD at set intervals to monitor the polymorph transformation.

### 2.7. Computational methods   

#### 2.7.1. Computation of molecular geometries and the potential energy surface   

Quantum mechanical calculations were performed using the *GAUSSIAN09* package (Frisch, 2009 ▸). Molecular models of diacids, including conformations in modification I and II, were retrieved from the experimental crystal structures. The potential energy surface (PES) of DA5 and DA11 about torsion angles τ_1_ and τ_2_ were generated in a vacuum and in solvent with τ_2_ constrained every 15° between −180 and 180° by optimizing the molecular geometries. The calculations were carried out using the SMD implicit solvation model for different solvents (ethanol and 1,4-dioxane) at the B97D/6–31+G (d,p) level, which has been widely verified (Du *et al.*, 2015 ▸; Khamar *et al.*, 2014 ▸; Grimme, 2006 ▸). Computed energetics and geometry optimizations at the B97D/6–31+G (d,p) level agreed extremely well with results computed by a double hybrid functional and a larger basis set (B2PLYD/def2QVZPP) (Du *et al.*, 2015 ▸).

#### 2.7.2. Computation of dimer geometries and energies   

Three types of carboxyl-dimer models of diacids were built based on crystal structures. Their geometry optimizations and frequency calculations were carried out in the gas phase and in two different SMD solvent models at the B97D/6–31+G (d,p) level of theory. Then *E*_e_ was re-computed via a single-point energy calculation of the optimized geometries with the same functional but a larger basis set (B97D/def2QZVPP). Use of large basis sets ensured that the basis set superposition error was minimized. The sum of the electronic energy (*E*_e_) plus the thermal free energy [*G*_corr(T)_], which was calculated from the frequency analysis, is the Gibbs free energy (*G*) [where *G*(T) = *E*_e_ + *G*_corr(T)_]. To minimize the computational cost while maintaining the accuracy, the *G*_corr(T)_ term was computed with the smaller basis set B97D/6–31+G(d,p) model. The free energies of three types of dimers were then calculated as the difference between the total free energy of the dimer (with conformer I or II) minus the free energy of two monomers with conformer I, which is the most stable based on our models. Similar computational models have recently been used to investigate self-association of various carboxylic acids in solution (Du *et al.*, 2015 ▸; Di Tommaso, 2013 ▸).

## 3. Results and discussion   

### 3.1. Polymorph screening and control of odd-carbon diacids by isothermal cooling experiments   

In our previous study on DA11 (Shi *et al.*, 2018 ▸), we concluded that form I (stable under ambient conditions) can generally be obtained from solvents with low HBD ability and form II is crystallized in solvents with high HBD ability. Here HBD and HBA abilities are represented by α and β, respectively.

**Figure 2:**
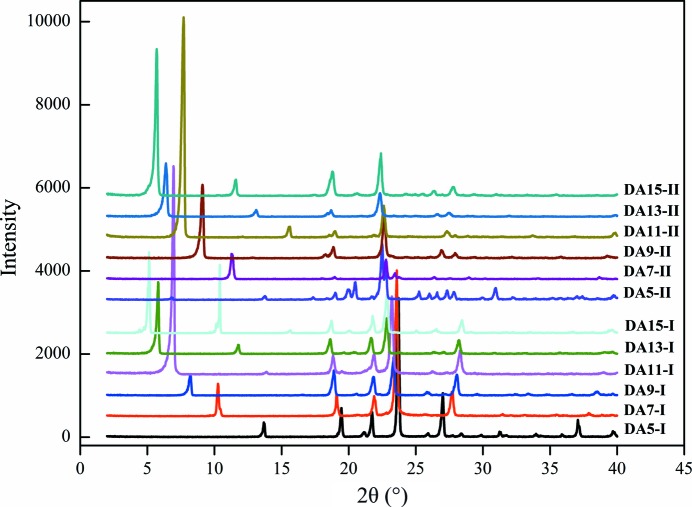
PXRD patterns of powder products of the six diacid forms.

Based on the results and their implications, attempts have been made to screen the polymorphs of DA15 and control the polymorphs of DA5–DA13 with various solvents and supersaturations. Apart from DA15, these diacids have been reported to crystallize in two forms (Shi *et al.*, 2018 ▸; Housty, 1968 ▸; Delarbre *et al.*, 1989 ▸; Kay & Katz, 1958 ▸; Thalladi *et al.*, 2000 ▸), and here we follow a consistent method for naming the low-temperature (ambient temperature) ‘form I’ and the high-temperature ‘form II’ (PXRD patterns are given in Fig. 2 ▸). One exception is the third form of DA7 (named γ), obtained by combined nanoscale crystallization (Ha *et al.*, 2009 ▸).

In this study, two solvents with high α values, ethanol and acetic acid, and two solvents with no HBD ability, ethyl acetate and 1,4-dioxane, were used to perform the nucleation experiments. The results are summarized in Table 1 ▸ with the corresponding PXRD spectra provided in Figs. S1–S6. As expected, two different forms of DA15, also named I and II, were obtained for the first time by solvent design; the corresponding PXRD patterns are shown in Fig. 2 ▸. Overall, similar results were obtained among these diacids and polymorph control was implemented. When *S* = 1.3, form I of the six diacids was obtained from ethyl acetate and 1, 4-dioxane. With higher supersaturation, the metastable form II of DA7–DA13 crystallized and mixed with form I in the products. Meanwhile, pure form II of DA7–DA15 can be harvested from ethanol and acetic acid regardless of the supersaturation. These results are in line with our expectations of high solvent-dependent polymorph selectivity.

However, it is strange that form II of DA5, which is known, did not appear in any of the solvent systems used. It seems to break the pattern of similarity of this homologous series in terms of solvent-dependent polymorph selectivity. Although polymorph control by solvent design based on the nucleation pathway of DA11 (Shi *et al.*, 2018 ▸) was not fully realized, the unexpected outcome drew our attention and might be a key probe to further understand conformational polymorph formation. The structural relevance between solution chemistry and molecular conformation, and the packing of solutes in the resultant crystals may still be relied upon. In fact, any difference in solutions or crystal structures might shed light on the reasons why the polymorph formation of DA5 is different from the others, and ultimately the polymorph nucleation mechanism itself.

### 3.2. Crystal structure analysis   

As expected, based on the PXRD patterns in Fig. 2 ▸, the crystal structure of different diacids should be similar or follow some rules. Those of short-chain dicarboxylic acids (DA5, DA7 and DA9) are mentioned in the literature (Thalladi *et al.*, 2000 ▸) and we have also reported for the first time the arrangement modes of two forms of long-chain diacids (DA11) (Shi *et al.*, 2018 ▸). With more information about the crystal structures of DA13 and DA15, here the structure regulations of six diacids (from DA5 to DA15), including the consistencies and differences, can be summarized and analyzed.

As shown in Tables S2 and S3, the crystal structures of two forms of DA5, DA7 and DA9 have been reported (Espeau *et al.*, 2013 ▸; Thalladi *et al.*, 2000 ▸; Bhattacharya *et al.*, 2013 ▸; Mishra *et al.*, 2015 ▸) and those of DA11 were also determined in our previous work (Shi *et al.*, 2018 ▸). Moreover, only one crystal structure of α (here named II) of DA13 was determined incompletely by two-dimensional X-ray diffraction (Housty, 1968 ▸). In fact, the longer the carbon chain, the more difficult it was to cultivate single crystals. For further comparison and analysis, we tried to obtain both crystal structures of two forms of DA13 and DA15. It is unfortunate that only single crystals of form II of the two compounds were obtained by slow evaporation of a formic−acetic acid mixture at 323 K, and both crystal structures were determined by SXRD; the crystallographic data are presented in Tables S4 and S5.

**Figure 3:**
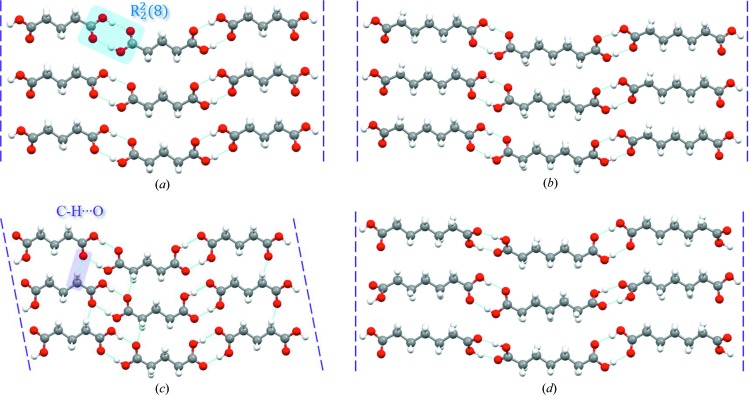
Neighboring hydrogen bond chains (*a*) in form I of DA5, (*b*) in form I of DA7, (*c*) in form II of DA5 and (*d*) in form II of DA7.

In fact, the dimorph structures of DA7, DA9, DA13 and DA15 and form I of DA5 are essentially in accordance with the characteristics of DA11, though the crystal structures of form I of DA13 and DA15 have still not been determined. The basic and unique synthon (Desiraju, 2013 ▸) is the carboxyl acid dimer shown in Fig. 3 ▸.

**Figure 4:**
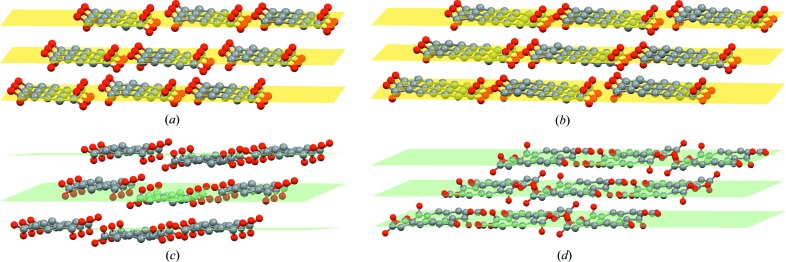
Layer stacking in crystals (*a*) in form I of DA5, (*b*) in form I of DA7, (*c*) in form II of DA5 and (*d*) in form II of DA7. Yellow and green bands represent the layers built by carboxyl chains. It is apparent that uneven stacking of layers exists in form II of DA5 compared with the others.

**Figure 5:**
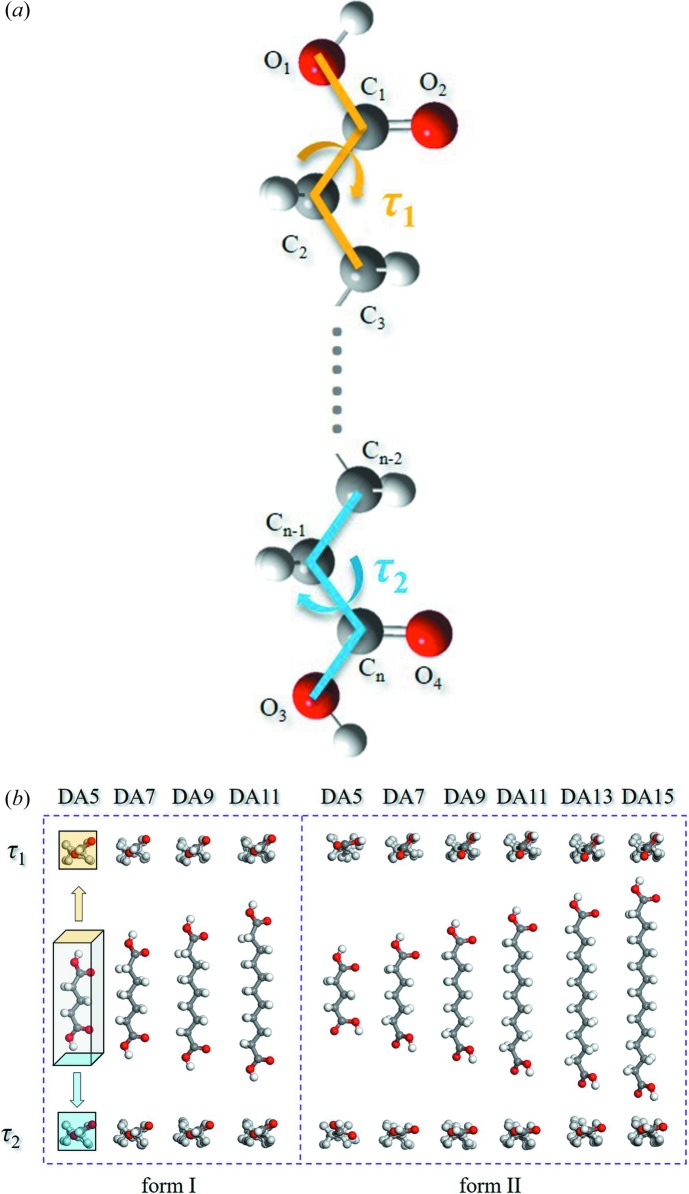
(*a*) Schematic of the twisting mode of the diacid molecules in crystals. The torsions at both ends (O_1_—C_1_—C_2_—C_3_ and O_3_—C_*n*_—C_*n*−1_—C_*n*−2_) are named τ_1_ and τ_2_, respectively. (*b*) Conformations in form I and II of the six diacids. Top and bottom views are shown for comparison of τ_1_/τ_2_ among the diacids.

Assembled molecular chains aggregate into a layer, and then into a crystal through hydrophobic interactions (Desiraju, 2001 ▸) as shown in Fig. 4 ▸. In order to pack as close as possible and adjust the distance between carboxyl dimers to keep the structure stable, the whole conformation twists in both forms, starting with carboxyl groups; twisting of other torsions is negligible. The torsions at both ends (O_1_—C_1_—C_2_—C_3_ and O_3_—C_*n*_—C_*n*−1_—C_*n*−2_) are named τ_1_ and τ_2_, respectively, and are shown schematically in Fig. 5 ▸(*a*). As shown in Fig. 5 ▸(*b*) and Table 2 ▸, the conformation in form I is molecular symmetry related thanks to τ_1_ = τ_2_ = ±160° approximately at both ends. However, in the form II conformation, only one carboxyl has a sharp twist about τ_2_ = ±36° (τ_1_ = ±180°), leading to a loss of molecular symmetry. It is worth noting that although diacids do not contain a chiral center, their conformations are chiral. Because of the presence of the inversion center C in both polymorphs, each crystal structure comprises both enantiomers (hence the ± sign for τ_1_ and τ_2_).

As expected, form II of DA5 shows some distinctions in its arrangement from the others, as illustrated in Fig. 3 ▸. Carboxyl dimer chains are also formed. When molecular chains aggregate into a layer, weaker C—H⋯O interactions are present among the chains in addition to hydrophobic interactions. This results from molecular chain offset during assembly which has not been observed for other odd-number diacids. Meanwhile, asymmetric twisting of both carboxyls also exists in the molecular conformation of the DA5 II-modification, with τ_1_ = ±148° and τ_2_ = ±7°, which is similar to form II of the other diacids despite the different values. Stable assembly among chains, especially carboxyl dimers, is achieved by these two patterns, including twisting and a unique offset. In addition, adjacent layers are not arranged parallel through hydrophobic interactions, which is different from other diacids (Figs. 4 ▸ and 6 ▸).

Combined with the previous solution nucleation results, it is clear that nucleation and the crystal structure are closely correlated. We did not obtain form II of DA5, whose structure was indeed different, based on the solvent-dependence selectivity of other diacids. This raises the question: what is the pathway through which such similar molecules assemble into crystals in different modes? We think such connections and distinctions are precious for exploring the nucleation pathway for a family of molecules that are similar in structure.

In fact, it could be concluded that the main difference between the two crystal forms of the six diacids including DA5 is the molecular conformation. Therefore, revealing how the different conformations are formed in crystals is the key to understanding the nucleation pathway.

### 3.3. Relative stability of the conformations of odd-carbon diacids in different solvents   

**Figure 7:**
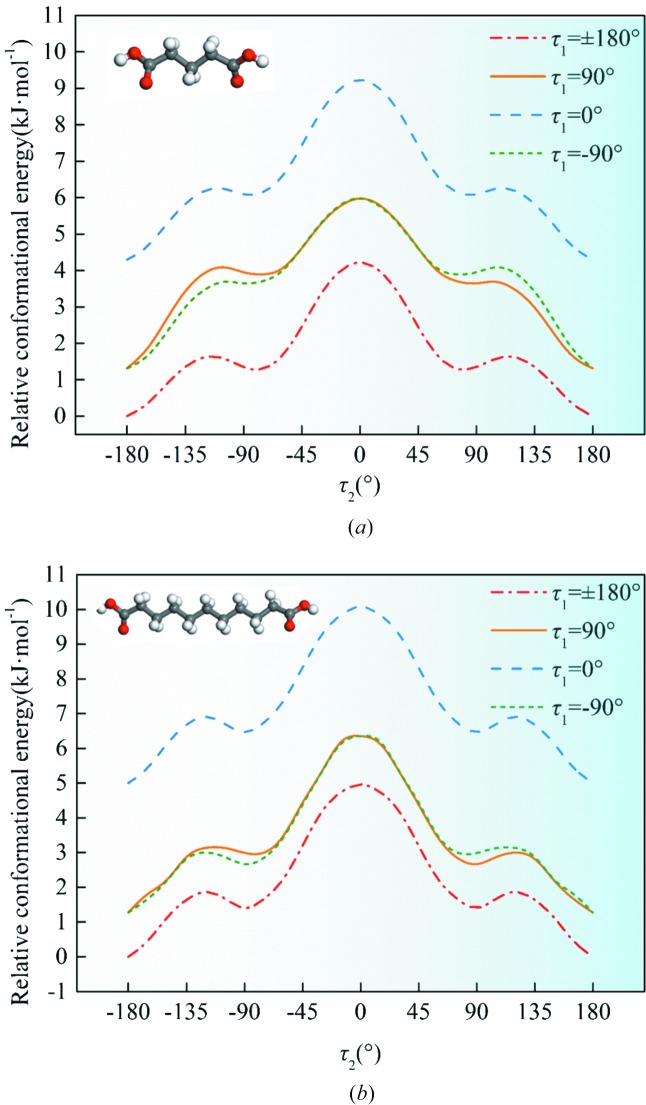
PES scans of (*a*) DA5 and (*b*) DA11 about the rotatable bond τ_2_ from −180 to 180° with τ_1_ fixed at different values in the gas phase.

**Figure 8:**
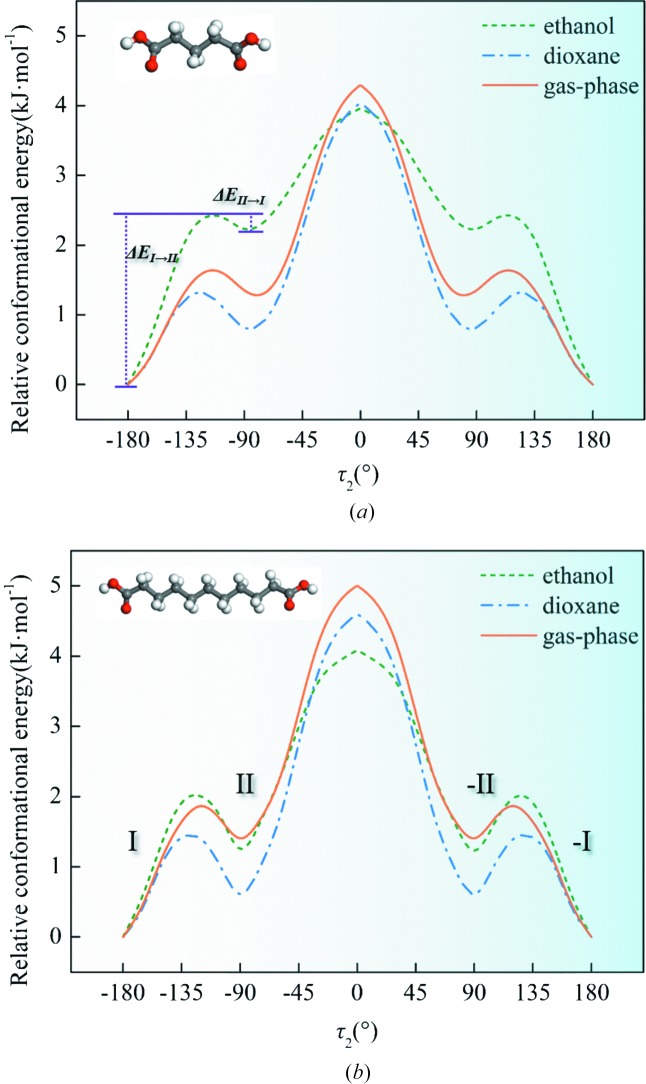
PES scans of (*a*) DA5 and (*b*) DA11 as a function of τ_2_ from −180 to 180° with τ_1_ = ±180° in the gas phase, in ethanol and 1,4-dioxane, respectively. Conformation I of both DA5 and DA11 are located in global minima and conformation II of both are situated in adjacent local minima. Δ*E*_I→II_ and Δ*E*_II→I_ are the energy barriers for the conformational change from I to II and II to I, respectively.

The relative stability of the diacid conformations is the key to understanding the role of conformational flexibility during crystallization. Firstly, molecular geometry optimization stated that the lowest-energy conformation in either gas or solvents was with τ_1_ = τ_2_ = ±180° and the values of the torsions formed by four neighboring C atoms were also ±180°. Based on the conclusion that τ_1_ and τ_2_ are the main torsion angles, we have computed the potential energy surface (PES) of DA5 and DA11, as a representative, about the rotatable bond τ_2_ with τ_1_ fixed at different values in gas and in two classes of solvents (ethanol with HBDs and 1,4-dioxane without HBDs). In Figs. 7 ▸ and S1, it is clear that τ_1_ and τ_2_ are almost independent of each other in DA5 and DA11 owing to the long carbon chains in various media. The most stable conformation is τ_1_ = τ_2_ = ±180°. Meanwhile, for all diacids, the value of τ_1_ in both conformations is close to ±180°. Fig. 8 ▸ shows the PES scans of DA5 and DA11 as a function of τ_2_ with τ_1_ = ±180° in different media. The PES distributions of two enantiomers are essentially symmetric. Therefore, we will only consider and discuss one of them (τ_2_ = −180–0°).

It is apparent, based on the torsions in Table 2 ▸, that conformations in form I and II are located in two adjacent local energy minimum regions, respectively, as showed in Fig. 8 ▸. Therefore, the dimorphism of odd-number diacids belongs to conformational polymorphism according to the concept raised by Aurora and coworkers (Cruz-Cabeza & Bernstein, 2014 ▸). The conformations of forms I and II are named conformation I (-I) and II (-II), respectively. Conformation I of the diacids is located in the global energy minimum region even though some conformational adjustment (no change) (Cruz-Cabeza & Bernstein, 2014 ▸) exists relative to the most stable conformation (τ_1_ = τ_2_ = ±180°), named conformer I. Meanwhile, conformer II is situated in the neighboring local energy minimum region with no conformational change (Cruz-Cabeza & Bernstein, 2014 ▸), where the conformation at the local minimum point is named conformer II. That is, conformations I and II are formed through conformational adjustment of conformers I and II, distinct energy minima of the gas-phase potential energy surface (Cruz-Cabeza & Bernstein, 2014 ▸). It is worth mentioning that, despite the different values of τ_1_ and τ_2_ in both forms of DA5 and other diacids, they are all located in the same energy-minimum region as shown in Fig. 8 ▸. The relative stability of conformers I and II of either DA11 or DA5 was considered constant in gas, ethanol and 1,4-dioxane. The energy barriers for the conversion of conformer I to II (Δ*E*_I→II_) and of conformer II to I (Δ*E*_II→I_) are about 1.5–2.5 and 0.5 kJ mol^−1^ in both solvents, respectively, which means the interconversion between the two conformers should be facile in both types of solvents, where there is no distinct difference between DA5 and the other diacids (Derdour & Skliar, 2014 ▸). Combined with a large number of examples of conformation analysis in solutions (Derdour & Skliar, 2014 ▸; Du *et al.*, 2015 ▸), our results here show that the conformation of these molecules with high flexibility is not fixed or selected in solution.

### 3.4. The solute species of odd-carbon diacids in different solvents   

Considering the crystal structures as guides with one type of functional group, carboxyl–carboxyl packing could be envisaged as the possible mode of diacid molecule self-assembly in solution. Here, solution IR of diacids at different concentrations in ethanol (α = 86) and 1,4-dioxane (α = 0) was employed to analyze solute self-assembly and solute–solvent interactions in solution (Tang *et al.*, 2017 ▸; Kulkarni *et al.*, 2012 ▸; Sun & Xue, 2015 ▸). It is worth noting that the IR spectra of the selected solvents do not interfere with those of the carboxyl.

**Figure 9:**
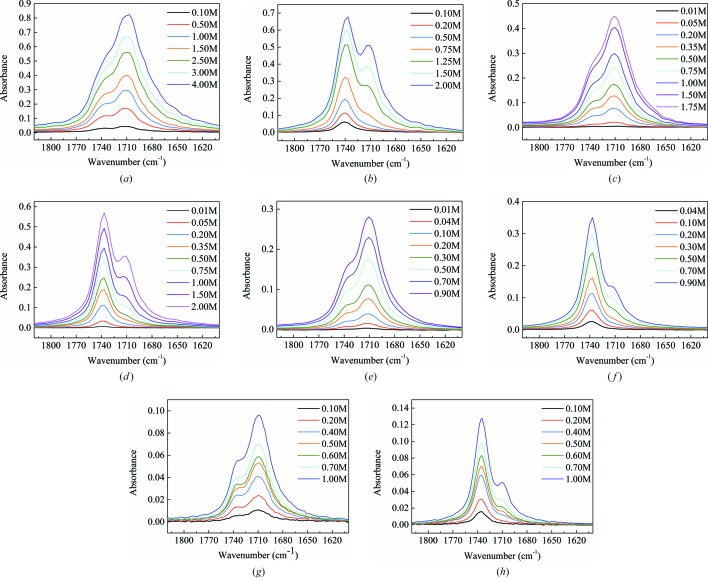
Solution IR spectra of diacids over a concentration range: (*a*) DA5 in ethanol, (*b*) DA5 in 1,4-dioxane, (*c*) DA7 in ethanol, (*d*) DA7 in 1,4-dioxane, (*e*) DA9 in ethanol, (*f*) DA9 in 1,4-dioxane, (*g*) DA11 in ethanol, (*h*) DA11 in 1,4-dioxane.

As shown in Fig. 9 ▸, there are two carboxyl bands at about 1736 and 1712 cm^−1^ in the IR spectrum of DA5/7/9/11 at low concentration in ethanol consistently. With the increasing concentration of diacids in ethanol, the intensity ratios of two bands remain constant and no obvious chemical shift occurs. It is evident that the solute species in ethanol with high HBD ability are unchanged as the concentration increases. Here we ascribe this difference to a solute–ethanol hydrogen-bond equilibrium [the low- and high-frequency C=O stretching frequencies corresponding to hydrogen-bonded and non-hydrogen-bonded C=O groups, respectively (Du *et al.*, 2015 ▸)]. Combined with the relevant literature (Du *et al.*, 2015 ▸, Tang *et al.*, 2017 ▸), there is another possibility. Diacid molecule–ethanol pairs are formed by hydrogen bonds between the carboxyl and hydroxyl groups, with ethanol acting as either an HBA (the higher frequency band) or an HBD (the lower frequency band). In any case, diacid molecules including DA5 and others are strongly solvated through hydrogen bonds without solute self-assembly in solvents with high *α.*

By contrast, in Fig. 9 ▸, only the peak at 1736 cm^−1^ is retained when the diacids are in 1,4-dioxane at low concentration, which should be the vibration band of the non-hydrogen-bonded C=O groups as the solvent has no HBDs. Surprisingly, with increasing concentration of DA5, DA7 and DA9 from unsaturation to saturation, a new band at about 1712 cm^−1^ appeared. The higher the concentration of the three diacids, the higher the intensity ratio of the band at 1712 cm^−1^ to that at 1736 cm^−1^. This supports that the carboxyl groups of diacids self-assemble in 1,4-dioxane after reaching a certain concentration, and the degree of aggregation increases with concentration (Kulkarni *et al.*, 2012 ▸; Khamar *et al.*, 2014 ▸), indicating intermolecular interaction of solutes rather than intramolecular interaction, *e.g.* molecule self-cyclization (Roux *et al.*, 2005 ▸). This differs greatly from solvents with high HBD ability where the diacid molecules exist as solvated or non-solvated monomers.

In addition, we found that the concentration where the band at 1712 cm^−1^ appears is almost concurrent at about 0.4–0.5 mol l^−1^ in three diacid solutions despite the fact that their solubility clearly decreases gradually as the carbon number increases (shown in Table S1). At which point, the IR spectra of the wider concentration range of DA11 in 1,4-dioxane from unsaturation to supersaturation were measured and indeed the band at 1712 cm^−1^ also appeared at about 0.4–0.5 mol l^−1^ which is supersaturated for DA11 at 298.15 K. This suggests that the absolute concentration, not solubility, of the two forms, has great effects on the solute self-assembly. In fact, there is strong experimental evidence for the molecular route of polymorph formation in our previous work (Shi *et al.*, 2018 ▸), where different solute species of DA11 (UDA) in different solvents were not detected. Instead, molecular dynamics calculations in dilute solution were conducted to validate that the solvation in ethanol and acetic acid is stronger than in solvents with no HBD ability. Here aggregates detected in 1,4-dioxane directly support that a lower degree of solvation exists in solvents with no HBD ability. This is a powerful experimental complement which shows the value of comparative study of a series of similar compounds. It could be concluded that no abrupt change about the solute species between DA5 and DA7 and others exists.

It is clear that there exists a significant difference in solute species in solvents with or without HBD ability. The absence of HBD ability of solvents provided more opportunities for solute self-aggregation. In view of the solvent dependence of polymorph nucleation and conformation as a key role in the formation of conformational polymorphism, it is naturally inferred that solute self-aggregation is closely related to configured conformation in the two forms.

### 3.5. The relationship between solute self-aggregation and conformations in polymorphs   

Here, additives were designed to disrupt solute self-aggregation in order to verify whether conformational polymorph formation would be influenced. In view of intermolecular interactions, different molecules (Fig. S8) with carboxyl groups were investigated as additives in 1,4-dioxane where only form I could be obtained initially and solute self-aggregation existed. The results for DA5-11 are shown in Table 3, ▸ which correspond to the PXRD patterns in Figs. S9–S16. We found that increasing the concentration of adipic acid (AA) contributed to the nucleation of form II of DA7, DA9 and DA11 but, unexpectedly, no changes in crystal nucleation occurred when adding benzoic acid (BA) even at high concentration. Additionally, these two additives both have no effect on the nucleation outcomes of DA5, which is still a valuable exception.

**Figure 10:**
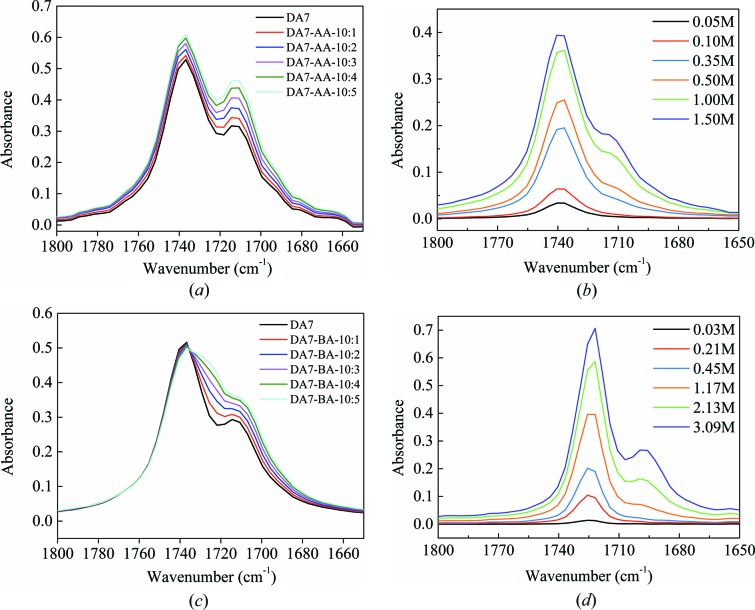
Solution IR spectra of additives in DA7 solution at the initial 1.75*M* in dioxane over a concentration range: (*a*) adipic acid, (*c*) benzoic acid. Solution IR spectra of additives in 1,4-dioxane over a concentration range: (*b*) adipic acid, (*d*) benzoic acid.

To evaluate how the additives work, solution FTIR experiments involving various concentrations of additives and DA7 (DA5) in dioxane have been conducted. First, the solution of DA7 (DA5) in dioxane at 1.75 mol l^−1^ was prepared (where the band of solute aggregations appears). Then additives AA or BA were added gradually (with a molar ratio of 1:10 additives to solute every time). The solution FTIR spectra of DA7 are shown in Fig. 10 ▸. It is evident that with adding AA for the first time (1:10), the peak intensity of solute aggregation at about 1712 cm^−1^ increases, even more than the growth of solvated monomers [Fig. 10 ▸(*a*)], meaning more intermolecular interactions between DA7–AA, AA–AA or both occur. For comparison, there is no obvious peak at a similar position in the spectra of pure AA dissolved in dioxane at a similar concentration, about 0.175 mol l^−1^ [Fig. 10 ▸(*b*)]. Hence, interactions forming between solute DA7 and AA are very likely. Upon adding more AA, the peak intensity at about 1712 cm^−1^ increases continuously. The interaction between DA7 and AA is formed, which modifies the nucleation pathway. When BA is the additive, the intensity of the peak positioned between the bands of monomers (1736 cm^−1^) and aggregations (1712 cm^−1^) increases gradually [Fig. 10 ▸(*c*)]. In fact, as shown by Fig. 10 ▸(*d*), the signal can be assigned to the BA monomers. To some extent, this interferes with our judgment about whether DA7–BA interactions occur or not. However, we can still say that an obvious signal to show whether BA can disturb the intermolecular interactions in DA7 solution, as in the case of AA, was not recognized.

Moreover, the IR spectra in Fig. 10 ▸(*b*) clearly show the band of carboxyl aggregation among AA in 1,4-dioxane at 1711–1714 cm^−1^, which is close to that of solute diacids. Thus the interaction between additive molecules is similar to that between solutes, and sufficient additives can disrupt solute self-aggregation. However, for benzoic acid, the band appears at 1696–1699 cm^−1^, meaning stronger interactions among these additive molecules which results in little disruption of solute self-aggregation, having no effect on polymorph nucleation. Given that conformation plays a key part in conformational polymorphism, this illustrates that solute self-aggregation is strongly associated with conformation in the two forms.

Similar results from IR were obtained in the case of DA5 shown in Fig. S17. That is, similar additive disruption in solutions of DA5 with DA7 occurred, but there were no changes in the nucleation outcomes. This further supports that there is no difference between the solute species in solutions of DA5 and DA7 and others.

**Figure 11:**
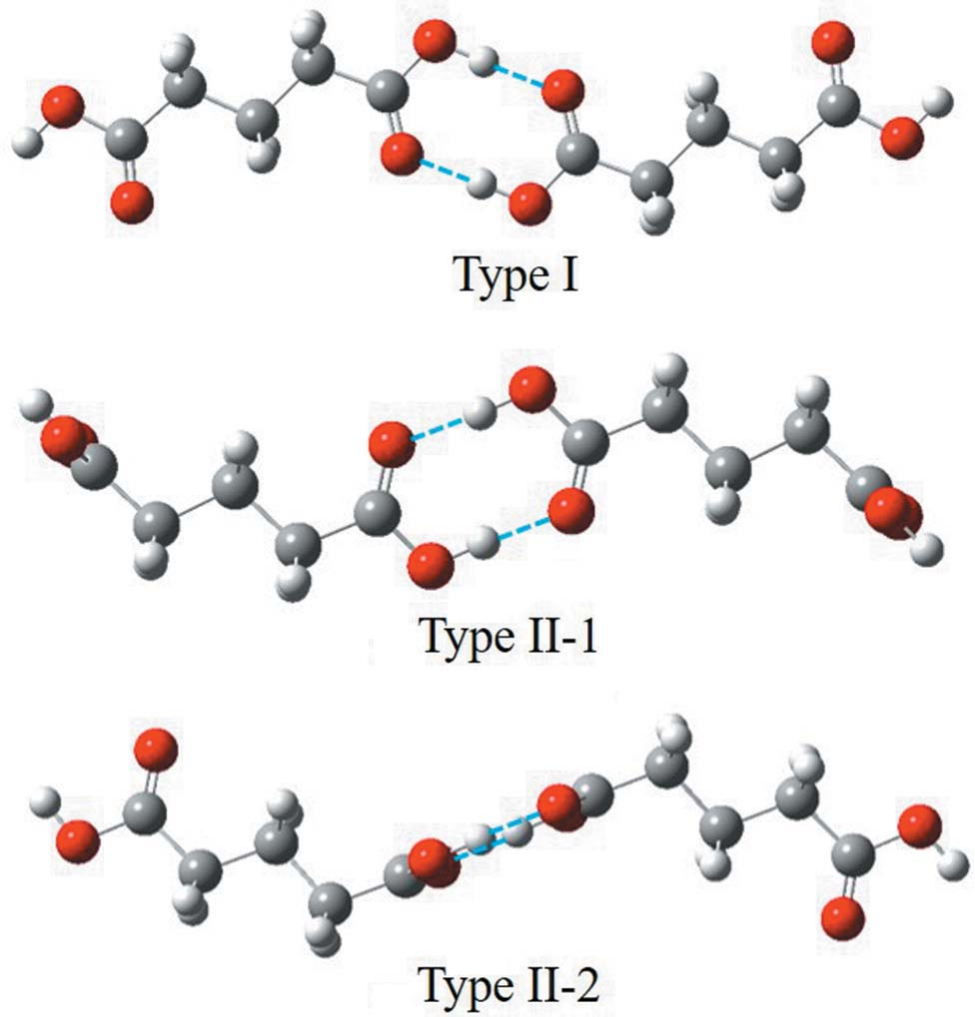
The three types of carboxyl dimer models of DA5.

Although it was proven that the solvent environment itself is not selective for the conformer, there is a hypothesis that solute self-aggregation in solvents with no HBD ability could result in a ‘lock’ in conformation corresponding to form I, which has also been discussed for the polymorph formation of tolfenamic acid (Mattei & Li, 2012 ▸, Du *et al.*, 2015 ▸). As shown in Table 4 ▸, we computed the dimerization energies (0 K) and free energies (at 298.15 K) for three types of carboxyl dimers of DA5 (Fig. 11 ▸) and DA11 built from two conformers in different media. The first was from form I where the molecule conformation was symmetrical, named Type I. The second and third dimer models were built on the basis of conformation in asymmetry in form II, named Type II-1 (dimers of carboxyl 1) and Type II-2 (dimers of carboxyl 2), respectively.

Negative energies indicate that a dimer is more favoured than two monomers at 0 K in any medium. And at temperatures higher than 298.15 K, due to the entropic penalty with dimerization, the relative stability changes. In the gas phase, a dimer is still favored over monomers. In ethanol, monomers have a higher stability as expected, but in 1,4-dioxane, the free energy is close to zero which indicates that the two configurations show similar stability (Du *et al.*, 2015 ▸). This is consistent with the experimental results, showing that solute aggregations were detected in 1,4-dioxane with no HBD ability, but not in ethanol. With respect to the molecular conformation, the energy differences among three types of dimers with two conformers are small, so solute self-aggregations could not lead to conformation immobilization. As for both of the diacids, they follow similar patterns. Moreover, Table 1 ▸ shows that the metastable form II, in solvents with no HBD ability, is more favored under high supersaturation, however, with high degree of self-aggregation. Form II of DA5 has not been observed, although no obvious solute self-aggregation was determined in solvents with HBD. All this illustrates that solute self-aggregation is unlikely to be a cause of conformational restriction in solvents.

Overall, different conformational states are almost surely formed during the nucleation process, that is, conformational rearrangement is inevitable (Davey *et al.*, 2006 ▸). So, there must exist a strong link between solute self-aggregation/desolvation and conformational rearrangement, key steps in the overall nucleation. In solvents with no HBD ability, solute self-aggregations are formed before nucleation with weaker solvation effect. Thus the conformation changes to stable I. On the contrary, no solute self-aggregation occurs with a stronger solvation effect, and metastable conformer II was formed. Diacids, with the exception of DA5, follow this nucleation pathway.

We did not observe any difference in solution chemistry between DA5 and the other diacids. Therefore, once again, attention should be paid to crystal structures. We previously analyzed the tiny differences in the crystal assembly of DA5, and it was found that the difference in packing efficiency between form I and II (ΔPE_I-II_) of DA5 was significantly greater than those of other diacids, shown in Table 5 ▸. *M*, *Z* and *V* represent the molecular weight, number of molecules in a cell and cell volume, respectively. There should be a bigger difference between the stability of the two forms of DA5 than that of other diacids. Polymorph transformation experiments indicated that the complete transformation from II to I of DA5 would be completed within 1 h under ambient conditions. However, similar transformations of the other diacids did not occur after 15 d (Table 5 ▸). The distinctions and similarities of all the diacids in solution chemistry enlighten us to the fact that, during nucleation of the conformation, rearrangement is likely to occur in two steps: first to metastable conformation II, then to stable conformation I. Desolvation and solute aggregation are the only obstacles that must be overcome before these steps. Weak solvation results in solute desolvation and self-aggregation earlier, and the conformation sufficiently rearranges to stable form I. Conversely, harder desolvation in combination with a stronger solvation effect leads to no solute aggregation before nucleation, hence metastable II is favored to crystallize after insufficient conformation rearrangement. For DA5, owing to the large difference between the stability of the two forms, difficult desolvation does not restrict sufficient conformation rearrangement to stable I. It also follows that this nucleation pathway further supports the relationship among solute self-aggregation/desolvation, conformation rearrangement and polymorphic outcomes.

## 5. Conclusions   

For the study of polymorph nucleation of DA11, we employed various solvents with different HBD and HBA abilities in an attempt to realize polymorph control and screening of a series of materials (DA5, DA7, DA9, DA13 and DA15). In terms of results, most compounds (DA7–DA13) were precisely controlled in terms of polymorphic outcomes and the dimorphism of DA15 has come to light. DA5 was a surprising exception that provided a new perspective, that is, any difference from solution to crystal might be the reason for the different polymorphs formed for different materials, providing further insight into the nucleation pathway and mechanism.

In addition, it was concluded that conformational differences are the major contribution to distinguishing dimorphs with the aid of existing and new data for the crystal structures of six compounds. Thus, molecular conformation was regarded as another thread from solution to crystal. In this exploration, we discussed the comparisons of two dimensions, including two forms and six model materials, by combining experiments and calculations. Finally, a structural pathway involving desolvation/solute self-aggregation, which impacts the extent of conformational rearrangement and further influences the polymorphic outcomes, has been verified.

Compared with previous studies of nucleation pathways, here, contrast among six similar compounds was introduced. The connections could verify or rule out some possibilities and the distinctions help reveal the key step during the nucleation process. To some extent, it makes up for the lack of research techniques and the fragmentization of studied compounds, which are bottlenecks in the study of nucleation mechanisms. We believe that the results may shed some light on the role of solvation, structural rearrangement, packing and conformation during nucleation. On one hand, the raised route can guide us to control and screen polymorphs by changing the solvation/intermolecular interactions or kinetic factors. On the other hand, the possible relationship between polymorph nucleation, especially structural rearrangement and transformation, deserves further research.

## 4. Related literature   

The following references are cited in the supporting information: Dolomanov *et al.* (2009 ▸); Rigaku (2018 ▸). 

## Supplementary Material

Crystal structure: contains datablock(s) DA13, DA15. DOI: 10.1107/S205225252000233X/lq5027sup1.cif

Structure factors: contains datablock(s) DA15. DOI: 10.1107/S205225252000233X/lq5027sup2.hkl

Structure factors: contains datablock(s) DA13. DOI: 10.1107/S205225252000233X/lq5027sup3.hkl

Supporting information file. DOI: 10.1107/S205225252000233X/lq5027sup4.pdf

CCDC references: 1941171, 1941172

## Figures and Tables

**Figure 6:**
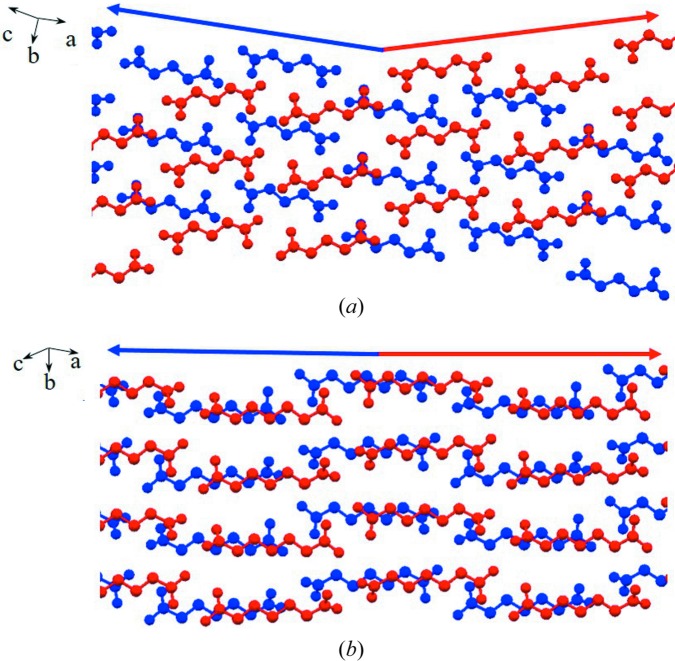
Layer stacking: (*a*) not parallel in form II of DA5 and (*b*) parallel in form I of DA7.

**Table 1:** Solvent property parameters and the forms obtained in different supersaturations

Solvent	α	β	π*	Diacid	Form (*S* = 1.3)	Form (*S* = 2.5)
Ethyl acetate†	00	45	55	DA5	I	I
				DA7	I	I+II
				DA9	I	I+II
				DA11	I	I+II
				DA13	I	I+II
				DA15	I	I+II
1,4-Dioxane†	00	37	55	DA5	I	I
				DA7	I	I+II
				DA9	I	I+II
				DA11	I	I+II
				DA13	I	I+II
				DA15	I	I+II
Ethanol†	86	75	54	DA5	I	I
				DA7	II	II
				DA9	II	II
				DA11	II	II
				DA13	II	II
				DA15	II	II
Acetic acid†	112	45	64	DA5	I	I
				DA7	II	II
				DA9	II	II
				DA11	II	II
				DA13	II	II
				DA15	II	II

† Solvent property parameters were obtained from the work by Marcus (1993 ▸).

**Table 2:** The torsions τ_1_ and τ_2_ of molecules in both forms of diacids

Form	Diacid	τ_1_(°)	τ_2_(°)	CCDC
I	DA5	−156.30 (156.30)	−156.30 (156.30)	1061299
	DA7	−162.99 (162.99)	−162.99 (162.99)	1233866
	DA9	−163.31 (163.31)	−163.31 (163.31)	1104213
	DA11	−163.14 (163.14)	−163.14 (163.14)	1841530
II	DA5	−148.44 (148.44)	−7.17 (7.17)	891106
	DA7	176.55 (−176.55)	−37.01 (37.01)	929796
	DA9	179.02 (−179.02)	−36.73 (36.73)	929807
	DA11	178.94 (−178.94)	−36.71 (36.71)	1841531
	DA13	179.07 (−179.07)	−36.69 (36.69)	1941171
	DA15	178.99 (−178.99)	−37.88 (37.88)	1941172

**Table 3:** The effects of additives on polymorph formation of DA7 in 1,4-dioxane

Diacid	Additive	Molar ratio (additive:solute)					
		1:10	2:10	3:10	4:10	5:10	1:1
DA5	Adipic acid	I	–	–	–	I	I
	Benzoic acid	I	–	–	–	I	I
DA7	Adipic acid	I+II	II	II	II	II	II
	Benzoic acid	I	–	–	–	I	I
DA9	Adipic acid	I	I+II	I+II	II	–	–
	Benzoic acid	I	I	I	I	–	I
DA11	Adipic acid	I+II	I+II	I+II	II	–	–
	Benzoic acid	I	I	I	I	–	I

**Table 4:** Dimerization energies (Δ*E*_d_) and free energies (Δ*G*_298.15 K_) for DA5 and DA11 in three types of carboxyl dimer in different media

Diacid	Conditions	Type	Δ*E*_d_ (kJ mol^−1^)	Δ*G*_298.15k_ (kJ mol^−1^)
DA5	Gas phase	I	−67.72	−12.01
		II-1	−65.03	−9.03
		II-2	−67.17	−9.90
	Ethanol	I	−35.46	14.32
		II-1	−31.75	17.33
		II-2	−33.42	24.27
	1,4-Dioxane	I	−57.49	4.58
		II-1	−55.77	1.64
		II-2	−57.64	0.98
DA11	Gas-phase	I	−67.04	−15.41
		II-1	−64.57	−12.82
		II-2	−66.88	−3.65
	Ethanol	I	−35.38	10.25
		II-1	−33.65	14.09
		II-2	−33.96	16.57
	1,4-Dioxane	I	−57.02	2.90
		II-1	−56.15	2.38
		II-2	−58.13	1.87

**Table 5:** Calculated packing efficiency and stability of different modifications of diacids under ambient conditions

Diacid	Form	*M*	*Z*	*V* (Å^3^)	PE†	ΔPE_I-II_ ‡	Stability	CCDC
DA5	I	132.11	4	595.2	0.8878	0.0749	>15 d	1061299
	II	132.11	8	1300.1	0.8129		<1 h	891106
DA7	I	160.17	4	775.656	0.8260	0.0301	>15 d	1233866
	II	160.17	4	805	0.7959		>15 d	929796
DA9	I	188.22	4	959.261	0.7849	0.0159	>15 d	1104213
	II	188.22	4	978.978	0.7690		>15 d	929807
DA11	I	216.27	4	1143.2	0.7567	0.0145	>15 d	1841530
	II	216.27	4	1165.6	0.7422		>15 d	1841531
DA13	II	244.33	4	1364.3	0.7164	–	>15 d	1941171
DA15	II	272.38	4	1508.8	0.7221	–	>15 d	1941172

† Packing efficiency, 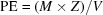.

‡ Difference in packing efficiency between forms I and II.

## References

[bb48] Back, K. R., Davey, R. J., Grecu, T., Hunter, C. A. & Taylor, L. S. (2012). *Cryst. Growth Des.* **12**, 6110–6117.

[bb49] Bhattacharya, S., Saraswatula, V. G. & Saha, B. K. (2013). *Cryst. Growth Des.* **13**, 3651–3656.

[bb3] Burton, R. C., Ferrari, E. S., Davey, R. J., Finney, J. L. & Bowron, D. T. (2010). *J. Phys. Chem. B*, **114**, 8807–8816. 10.1021/jp103099j 20550207

[bb4] Chadwick, K., Davey, R. J., Dent, G., Pritchard, R. G., Hunter, C. A. & Musumeci, D. (2009). *Cryst. Growth Des.* **9**, 1990–1999.

[bb5] Cruz-Cabeza, A. J. & Bernstein, J. (2014). *Chem. Rev.* **114**, 2170–2191. 10.1021/cr400249d 24350653

[bb7] Davey, R. J. (2004). *Nature*, **428**, 374–375. 10.1038/428374a 15042070

[bb6] Davey, R. J., Dent, G., Mughal, R. K. & Parveen, S. (2006). *Cryst. Growth Des.* **6**, 1788–1796.

[bb8] Davey, R. J., Schroeder, S. L. & ter Horst, J. H. (2013). *Angew. Chem. Int. Ed.* **52**, 2166–2179. 10.1002/anie.201204824 23307268

[bb9] Delarbre, J. L., Fabrègue, E., Maury, L. & Bardet, L. (1989). *J. Raman Spectrosc.* **20**, 41–48.

[bb10] Derdour, L. & Skliar, D. (2014). *Chem. Eng. Sci.* **106**, 275–292.

[bb11] Desiraju, G. R. (1997). *Science*, **278**, 404–405.

[bb12] Desiraju, G. R. (2001). *Nature*, **412**, 397–400. 10.1038/35086640 11473302

[bb13] Desiraju, G. R. (2013). *J. Am. Chem. Soc.* **135**, 9952–9967. 10.1021/ja403264c 23750552

[bb14] Desiraju, G. R. (2014). *IUCrJ*, **1**, 380–381. 10.1107/S2052252514021976 PMC4224455 25485117

[bb15] Di Tommaso, D. (2013). *CrystEngComm*, **15**, 6564–6577.

[bb104] Dolomanov, O. V., Bourhis, L. J., Gildea, R. J., Howard, J. A. K. & Puschmann, H. (2009). *J. Appl. Cryst.* **42**, 339–341.

[bb16] Du, W., Cruz-Cabeza, A. J., Woutersen, S., Davey, R. J. & Yin, Q. (2015). *Chem. Sci.* **6**, 3515–3524. 10.1039/c5sc00522a PMC5814770 29511513

[bb60] Espeau, P., Négrier, P. & Corvis, Y. (2013). *Cryst. Growth Des.* **13**, 723–730.

[bb18] Frisch, M. J. *et al.* (2009). *GAUSSIAN09*, Gaussian, Inc., Wallingford, CT, USA.

[bb19] Grimme, S. (2006). *J. Comput. Chem.* **27**, 1787–1799. 10.1002/jcc.20495 16955487

[bb20] Ha, J., Hamilton, B. D., Hillmyer, M. A. & Ward, M. D. (2009). *Cryst. Growth Des.* **9**, 4766–4777.

[bb21] Hollingsworth, M. D. (2002). *Science*, **295**, 2410–2413. 10.1126/science.1070967 11923527

[bb65] Housty, J. (1968). *Acta Cryst.* B**24**, 486–494.

[bb23] Ischenko, V., Englert, U. & Jansen, M. (2005). *Chem. Eur. J.* **11**, 1375–1383. 10.1002/chem.200400728 15619726

[bb24] Kay, M. I. & Katz, L. (1958). *Acta Cryst.* **11**, 289–294.

[bb25] Khamar, D., Zeglinski, J., Mealey, D. & Rasmuson, A. C. (2014). *J. Am. Chem. Soc.* **136**, 11664–11673. 10.1021/ja503131w 25029039

[bb26] Kulkarni, S. A., McGarrity, E. S., Meekes, H. & ter Horst, J. H. (2012). *Chem. Commun.* **48**, 4983–4985. 10.1039/c2cc18025a 22498662

[bb71] Kulkarni, S. A., Meekes, H. & ter Horst, J. H. (2014). *Cryst. Growth Des.* **14**, 1493–1499.

[bb28] Kulkarni, S. A., Weber, C. C., Myerson, A. S. & ter Horst, J. H. (2014). *Langmuir*, **30**, 12368–12375. 10.1021/la5024828 25256225

[bb29] Li, Z., Shi, P., Yang, Y., Sun, P., Wang, Y., Xu, S. & Gong, J. (2019). *CrystEngComm*, **21**, 3731–3739.

[bb74] Marcus, Y. (1993). *Chem. Soc. Rev.* **22**, 409.

[bb31] Mattei, A. & Li, T. (2012). *Pharm. Res.* **29**, 460–470. 10.1007/s11095-011-0574-7 21879384

[bb32] Mishra, M. K., Ramamurty, U. & Desiraju, G. R. (2015). *Chem. Asian J.* **10**, 2176–2181. 10.1002/asia.201500322 25919633

[bb33] Myerson, A. S. & Trout, B. L. (2013). *Science*, **341**, 855–856. 10.1126/science.1243022 23970690

[bb34] Parveen, S., Davey, R. J., Dent, G. & Pritchard, R. G. (2005). *Chem. Commun.* 1531–1533. 10.1039/b418603f 15770249

[bb82] Rath, N. P., Kumar, V. S. S., Janka, M. & Anderson, G. K. (2007). *Inorg. Chim. Acta*, **360**, 2997–3001.

[bb100] Rigaku (2013). *CrystalClear*. Rigaku AXS Inc., Toyko, Japan.

[bb105] Rigaku (2018). *CrysAlis PRO.* Rigaku Inc., Tokyo, Japan.

[bb36] Roux, M. V., Temprado, M. & Chickos, J. S. (2005). *J. Chem. Thermodyn.* **37**, 941–953.

[bb101] Sheldrick, G. (2014). *SHELXS2014*. Universität of Göttingen, Germany.

[bb102] Sheldrick, G. M. (2015). *Acta Cryst.* C**71**, 3–8.

[bb37] Shi, P., Ma, Y., Han, D., Du, S., Zhang, T. & Li, Z. (2019). *J. Mol. Liq.* **283**, 584–595.

[bb86] Shi, P., Xu, S., Du, S., Rohani, S., Liu, S., Tang, W., Jia, L., Wang, J. & Gong, J. (2018). *Cryst. Growth Des.* **18**, 5947–5956.

[bb39] Sun, C. & Xue, D. (2015). *CrystEngComm*, **17**, 2728–2736.

[bb40] Tang, W., Dai, H., Feng, Y., Wu, S., Bao, Y., Wang, J. & Gong, J. (2015). *J. Chem. Thermodyn.* **90**, 28–38.

[bb41] Tang, W., Mo, H., Zhang, M., Parkin, S., Gong, J., Wang, J. & Li, T. (2017). *J. Phys. Chem. B*, **121**, 10118–10124. 10.1021/acs.jpcb.7b07763 29017013

[bb42] Thalladi, V. R., Nüsse, M. & Boese, R. (2000). *J. Am. Chem. Soc.* **122**, 9227–9236.

[bb43] Xu, S., Wang, J., Zhang, K., Wu, S., Liu, S., Li, K., Yu, B. & Gong, J. (2016). *Chem. Eng. Sci.* **155**, 248–257.

[bb44] Yamasaki, R., Tanatani, A., Azumaya, I., Masu, H., Yamaguchi, K. & Kagechika, H. (2006). *Cryst. Growth Des.* **6**, 2007–2010.

[bb45] Zeng, Q., Mukherjee, A., Müller, P., Rogers, R. D. & Myerson, A. S. (2018). *Chem. Sci.* **9**, 1510–1520. 10.1039/c7sc04353h PMC5887107 29675194

[bb46] Zhang, H., Yin, Q., Liu, Z., Gong, J., Bao, Y., Zhang, M., Hao, H., Hou, B. & Xie, C. (2014). *J. Chem. Thermodyn.* **77**, 91–97.

[bb47] Zuñiga, F. J., Cruz-Cabeza, A. J., Aretxabaleta, X. M., de la Pinta, N., Breczewski, T., Quesada-Moreno, M. M., Avilés-Moreno, J. R., López-González, J. J., Claramunt, R. M. & Elguero, J. (2018). *IUCrJ*, **5**, 706–715. 10.1107/S2052252518011685 PMC6211527 30443355

