# Supplementary material for: Probing the structural pathway of conformational polymorph nucleation by comparing a series of α,ω-alkanedicarboxylic acids
Source: IUCrJ. 2020 Mar 26;7(Pt 3):422–33. doi: 10.1107/S205225252000233X (PMC7201272; doi:10.1107/S205225252000233X)
Supplement: Supplementary file 4 [file m-07-00422-sup4.pdf]

IUCrJ

Volume 7 (2020)

Supporting information for article:

Probing the structural pathway of conformational polymorph nucleation: by comparing a series of α,ω-alkanedicarboxylic acids

Peng Shi, Shijie Xu, Yiming Ma, Weiwei Tang, Feng Zhang, Jingkang Wang and Junbo Gong

The solubility measurement methods and results are shown in Table S1. Table S2 and S3 showed the crystallographic data of form I and form II of 6 diacids used in this work, respectively. Table S4 and S5 showed Experimental details of new structures of DA13 and DA15. PES scan of DA5 and DA11 about the rotatable bond τ_2 from -180° to 180° with τ_1 fixed at different values in solvents was presented in Fig. S1. The molecular structures of additives used in this study were shown as Fig. S2.

CCDC **1941171−1941172** (for the structures of form II of DA13 and DA15) contain the supplementary crystallographic data for this paper.

S1. Solubility Measurements

A gravimetric method was employed to determine the solubility of the stable form I of diacids in some mono-solvents (showed in Table S1) at 298.15 K. In this process, excess solid diacids and corresponding single, were added to 50 mL flasks so that to obtain the suspensions. Then the suspensions were shaken by a thermostatic bath shaker (CHY1015, Shanghai Sunny Hengping Scientific Instrument Co. Ltd., China) at a certain temperature under uncertainty of 0.1 K. And this process would last for 12 h which had been proved to be long enough to achieve solid-liquid equilibrium in preliminary experiment. After turning off the bath shaker, 5 mL of the supernatant liquor was filtered by the pre-cooled/heated syringes filters (0.22 μm) and moved into pre-weighted glass dishes as quickly as possible. Immediately, the total weight was determined. After that, the dishes were dried in a vacuum oven (DZ-2BC, Tianjin Taisite Instrument Co. Ltd., China) at T=343.15 K and their mass was periodically measured until the data remained constant, which meant that the solvent had been completely evaporated. In all above experiments, the masses were determined by an electronic balance (AB204-N, Mettler-Toledo, Switzerland) with an accuracy of ± 0.0001 g. The experiment was repeated three times for error reduction, and the result was from the average value.

The solubility of metastable form II of diacids was determined by dynamic method using the laser monitoring observation technique. At a given temperature, a fixed mass of solvent was added in the vessel with stirring. To avoid the solvent losses due to evaporation, a condenser was connected to the vessel. After the temperature of vessel was stable, a fixed amount of form II was added in the vessel. When the solid in the vessel disappeared completely, and the intensity of the transmitted laser through the solution reached the maximal value, the maximal value of the intensity was recorded as I_{max}. Then a small amount of form II which was accurately weighed was put into the vessel, after which the intensity of the transmitted laser decreased immediately. However, if the solution is unsaturated, the new added solid will dissolve gradually, and the maximum intensity of transmitted laser will be

reached at the end. The same procedure was repeated until the solution reached the saturation point. Then the total mass of form II added to the vessel was recorded and the solubility can be calculated. The interval of every addition was 60 min. For each measuring point, the same experiment was carried out three times.

The mole fraction solubility of diacids (x_1) was calculated by using Eq. (S1):

$$x_1 = \frac{m_1/M_1}{m_1/M_1 + m_2/M_2} \tag{S1}$$

Where m_1 represents the mass of solute diacids, m_2 mean the masses of solvents. M_1 and M_2 are the corresponding molecule mass of them.

The solubility of DA5, DA7, DA9, DA11, DA13 and DA15 (form I) and those of DA7, DA9, DA11, DA13 and DA15 (form II) in 4 mono-solvents was shown in **Table S1**.

Table S1 Molar solubility of diacids in solvents at 298.15 K.

Solvent	diacid	Molar solubility(form I)	Molar solubility(form II)
ethyl acetate	DA5	0.123[a]	-
	DA7	0.0517[a]	0.0541
	DA9	0.0161[a]	0.0168
	DA11	0.00456	0.00466
	DA13	0.00149[b]	0.00152
	DA15	0.00105	0.00108
1,4-dioxane	DA5	0.304	-
	DA7	0.138	0.155
	DA9	0.0823	0.0902
	DA11	0.0234	0.0263
	DA13	0.0151	0.0165
	DA15	0.00680	0.00762
ethanol	DA5	0.176[a]	-
	DA7	0.104[a]	0.120
	DA9	0.0707[a]	0.0775
	DA11	0.0273	0.0321

	DA13	0.00441[b]	0.00502
	DA15	0.00372	0.00435
acetic acid[a]	DA5	0.221[a]	-
	DA7	0.0964[a]	0.105
	DA9	0.0524[a]	0.0583
	DA11	0.0257	0.0285
	DA13	0.00418[b]	0.00448
	DA15	0.00134	0.00150

[a] Reference(Zhang *et al.*, 2014)is the source of data .

[b] Reference(Tang *et al.*, 2015) is the source of data.

Table S2 Crystallographic data of form I of 6 diacids.

	DA5-I [a]	DA7-I [b]	DA9-I [b]	DA11-I [c]
Formula	$C_5H_8O_4$	$C_7H_{12}O_4$	$C_9H_{16}O_4$	$C_{11}H_{20}O_4$
Crystal system			monoclinic	
Space group	*C*2/*c*	*C*2/*c*	*C*2/*c*	*C*2/*c*
T(K)	150(2)	130	130	133(2)
a(Å)	12.964(18)	17.7028(9)	22.6366(1)	26.597(7)
b(Å)	4.758(7)	4.7270(2)	4.7143(1)	4.7030(11)
c(Å)	9.747(14)	9.6713(4)	9.6162(3)	9.604(3)
β(°)	98.12(2)	106.580(1)	110.809(2)	107.899(4)
Cell volume(Å^3)	595.2(15)	775.656	959.261	1143.2(5)
Density(g/cm^3)	1.474	1.372	1.303	1.257
Z	4	4	4	4
R_{int}	0.0389	-	-	0.0344
R_1(I＞2sigma(I))	0.0376	0.0507	0.0454	0.0309
CCDC	1061299	1233866	1104213	1841530

[a] Reference (Mishra *et al.*, 2015) is the source of data.

[b] Reference (Thalladi *et al.*, 2000) is the source of data.

[c] Reference (Shi *et al.*, 2018) is the source of data.

Table S3 Crystallographic data of form II of 6 diacids.

	DA5-II [a]	DA7-II [b]	DA9-II [b]	DA11-II [c]	DA13-II	DA15-II
Formula	$C_5H_8O_4$	$C_7H_{12}O_4$	$C_9H_{16}O_4$	$C_{11}H_{20}O_4$	$C_{13}H_{24}O_4$	$C_{15}H_{28}O_4$
Crystal system			monoclinic			
Space group	*C*2/*c*	*P*2₁/*c*	*P*2₁/*c*	*P*2₁/*c*	*P*2₁/*n*	*P*2₁/*c*
T(K)	348	204	120	133	133	128
a(Å)	25.593	5.5593	5.5124	5.5078	5.5195	5.4671
b(Å)	5.0024	9.5787	9.4609	9.4058	9.4058	9.2806
c(Å)	10.1667	15.1193	18.8726	22.554	26.2830	29.8270
β(°)	92.740	90.972	95.932	94.018	90.84	94.449
Cell volume($Å^3$)	1300.1	805	978.978	1165.6	1364.3	1508.80
Density(g/cm^3)	1.355	1.313	1.277	1.232	1.189	1.199
Z	8	4	4	4	4	4
R_{int}	0.0719	0.0502	0.0774	0.0421	0.0811	0.0691
R_1(I＞2sigma(I))	-	0.0386	0.0689	0.0326	0.0575	0.0483
CCDC	891106	929796	929807	1841531	1941171	1941172

[a] Reference (Espeau *et al.*, 2013) is the source of data.

[b] Reference (Bhattacharya *et al.*, 2013) is the source of data.

[c] Reference (Shi *et al.*, 2018) is the source of data.

Table S4 Experimental details of DA13

Crystal data	
Chemical formula	$C_{13}H_{24}O_4$
M_r	244.32
Crystal system, space group	Monoclinic, $P2_1/n$
Temperature (K)	113
a, *b*, *c* (Å)	5.5195 (11), 9.4058 (19), 26.283 (5)

β (°)	90.84 (3)
V (Å^3)	1364.3 (5)
Z	4
Radiation type	Mo $K\alpha$
μ (mm^{-1})	0.09
Crystal size (mm)	0.20 × 0.18 × 0.15
Data collection	
Diffractometer	Rigaku Saturn 70 CCD
Absorption correction	Multi-scan *CrystalClear*(Rigaku,2008)
T_{min}, T_{max}	0.764, 1
No. of measured, independent and observed [$I > 2\sigma(I)$] reflections	12368, 3215, 2511
R_{int}	0.053
(sin θ/λ)$_{max}$ (Å^{-1})	0.658
Refinement	
$R[F^2 > 2\sigma(F^2)]$, $wR(F^2)$, S	0.057, 0.235, 1.03
No. of reflections	3215
No. of parameters	156
H-atom treatment	H-atom parameters constrained
$\Delta\rho_{max}$, $\Delta\rho_{min}$ (e Å^{-3})	0.47, −0.50

Computer programs: *CrystalClear* (Rigaku Inc., 2008), *ShelXT* (Sheldrick, 2015), *SHELXL* (Sheldrick, 2015), *Olex2* (Dolomanov *et al.*, 2009)

Table S5 Experimental details of DA15

Crystal data	
Chemical formula	$C_{15}H_{28}O_4$

M_r	272.37
Crystal system, space group	Monoclinic, $P2_1/c$
Temperature (K)	128
a, *b*, *c* (Å)	5.4671 (3), 9.2806 (5), 29.8270 (14)
β (°)	94.449 (4)
V (Å^3)	1508.80 (14)
Z	4
Radiation type	Mo *K*α
μ (mm^{-1})	0.09
Crystal size (mm)	0.34 × 0.26 × 0.16
Data collection	
Diffractometer	Rigaku Saturn 70
Absorption correction	Multi-scan<br>*CrysAlis PRO* 1.171.39.46 (Rigaku Oxford Diffraction, 2018) Empirical absorption correction using spherical harmonics, implemented in SCALE3 ABSPACK scaling algorithm.
T_{min}, T_{max}	0.787, 1.000
No. of measured, independent and observed [$I > 2\sigma(I)$] reflections	14484, 3599, 2690
R_{int}	0.052
$(\sin \theta/\lambda)_{max}$ (Å^{-1})	0.658
Refinement	
$R[F^2 > 2\sigma(F^2)]$, $wR(F^2)$, S	0.048, 0.130, 1.04
No. of reflections	3599
No. of parameters	180

H-atom treatment	H atoms treated by a mixture of independent and constrained refinement
$\Delta\rho_{max}$, $\Delta\rho_{min}$ (e Å^{-3})	0.27, −0.20

Computer programs: *CrystalClear* (Rigaku,2008), *CrysAlis PRO* 1.171.39.46 (Rigaku OD, 2018), ShelXT (Sheldrick, 2015), *SHELXL* (Sheldrick, 2015), Olex2 (Dolomanov *et al.*, 2009).

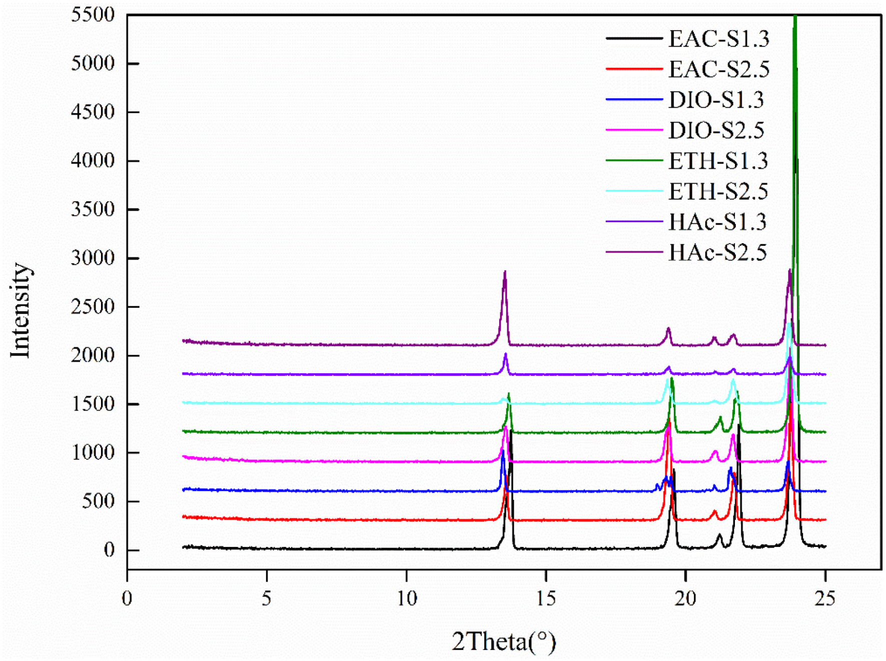

Figure S1 PXRD patterns of DA5 for experiments in Table 1.

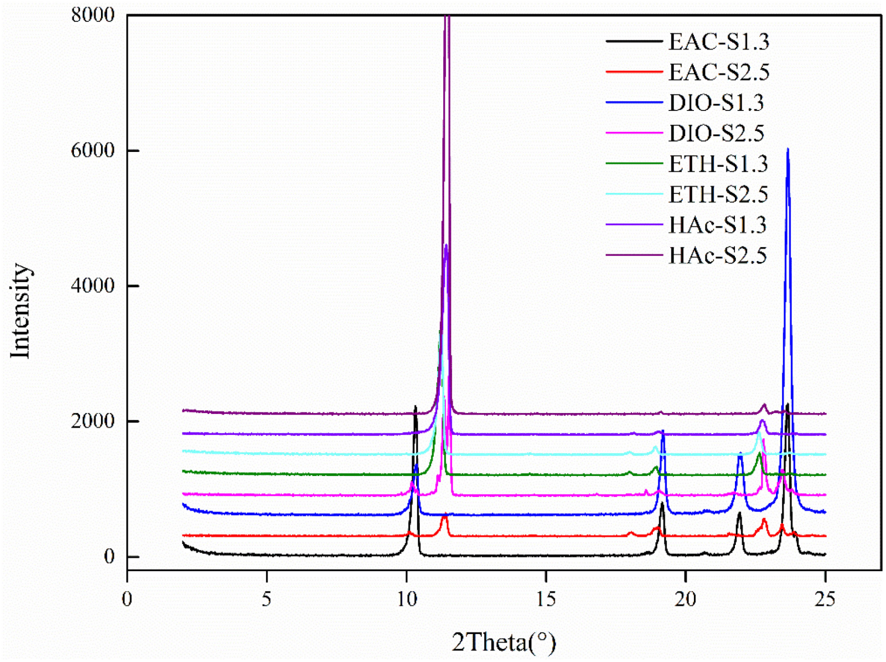

Figure S2 PXRD patterns of DA7 for experiments in Table 1.

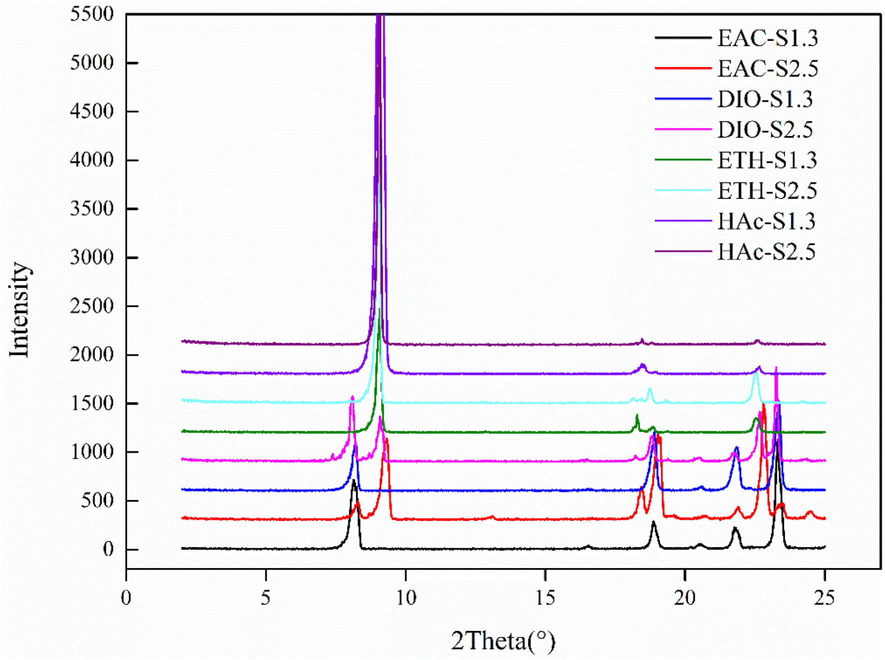

Figure S3 PXRD patterns of DA9 for experiments in Table 1.

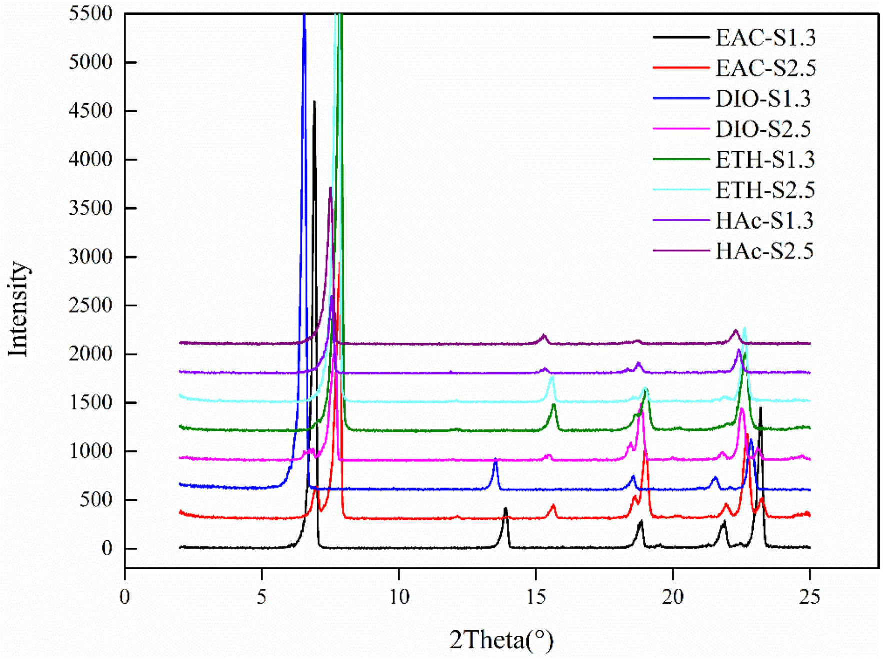

Figure S4 PXRD patterns of DA11 for experiments in Table 1.

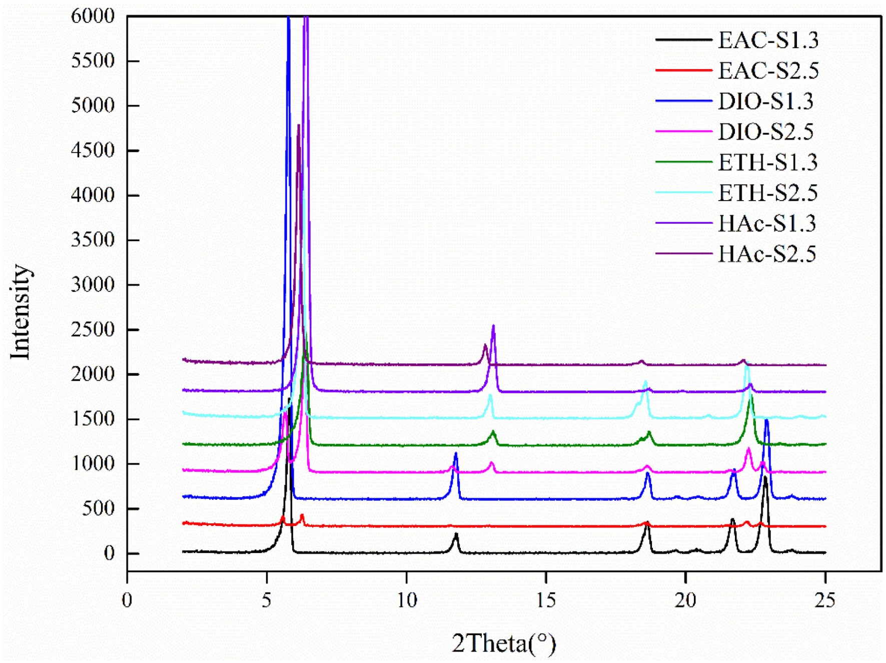

Figure S5 PXRD patterns of DA13 for experiments in Table 1.

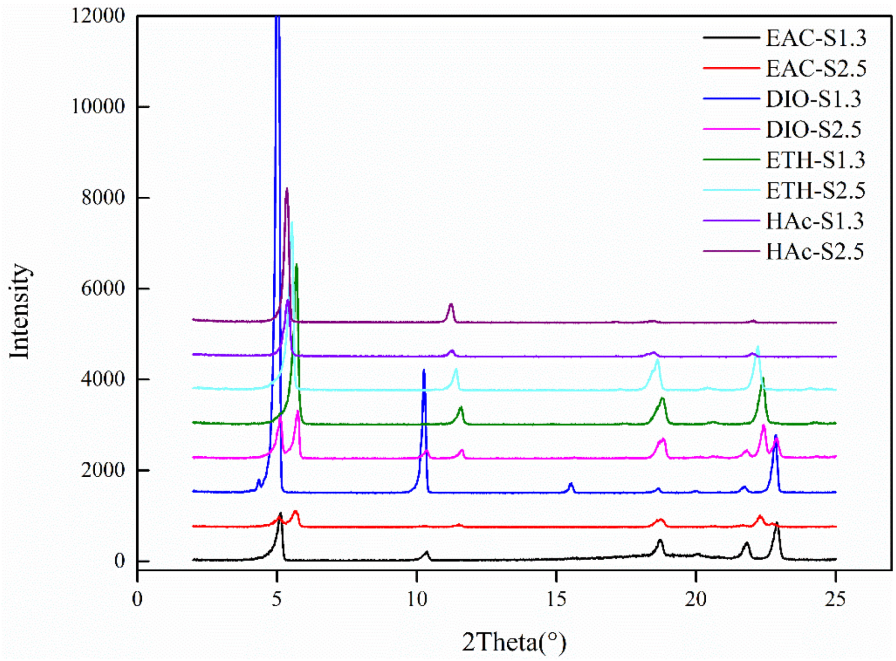

Figure S6 PXRD patterns of DA15 for experiments in Table 1.

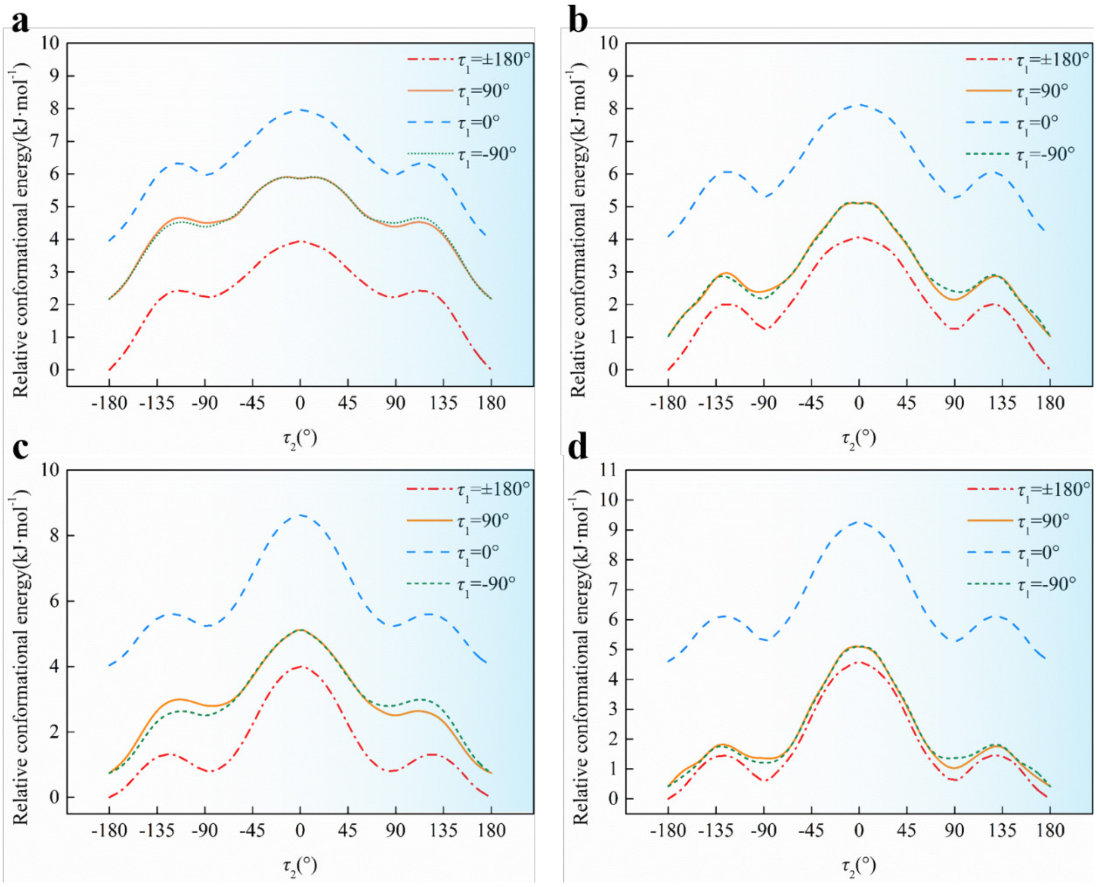

Figure S7 PES scan of DA5 and DA11 about the rotatable bond τ2 from -180° to 180° with τ1 fixed at different values in solvents. (a) DA5 in ethanol; (b) DA11 in ethanol; (c) DA5 in 1,4-dioxane; (d) DA11 in 1,4-dioxane.

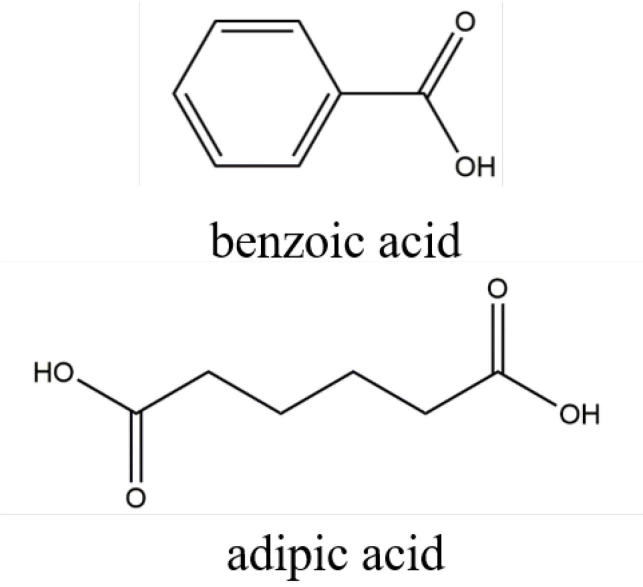

Figure S8 The molecular structures of additives.

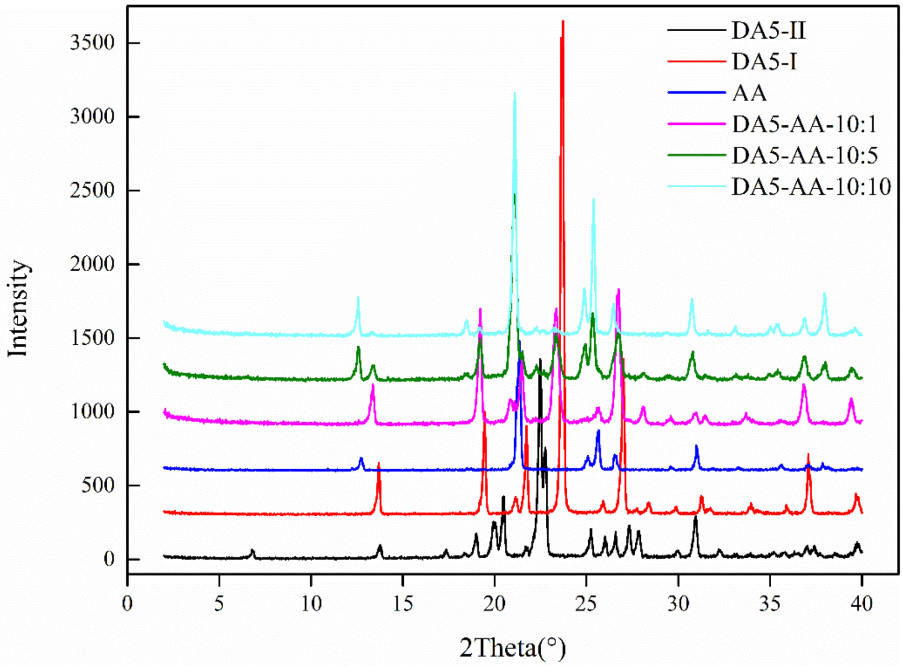

Figure S9 PXRD patterns of DA5 for additive experiments in Table 3(AA as additives)

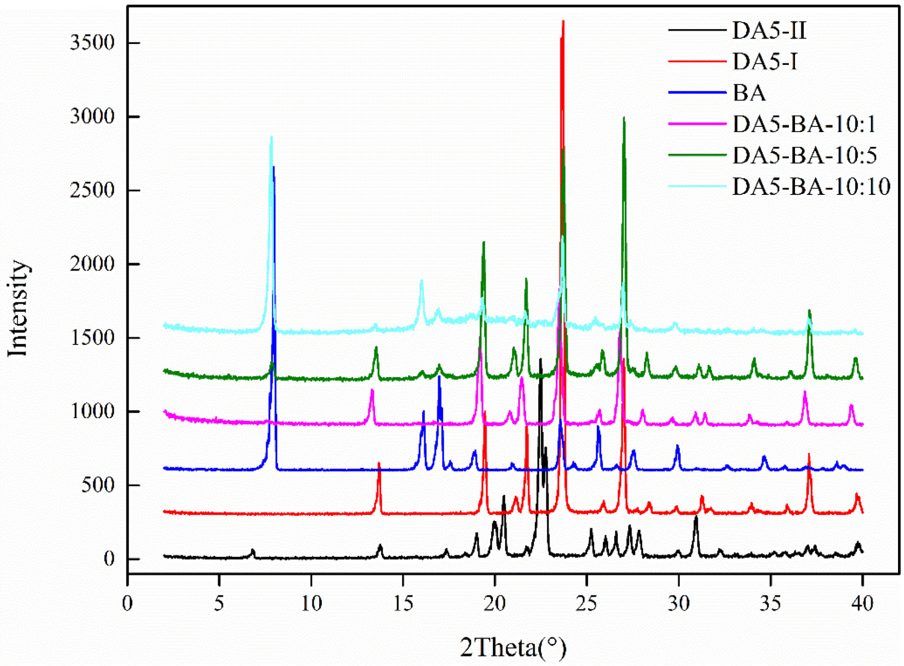

Figure S10 PXRD patterns of DA5 for additive experiments in Table 3(BA as additives)

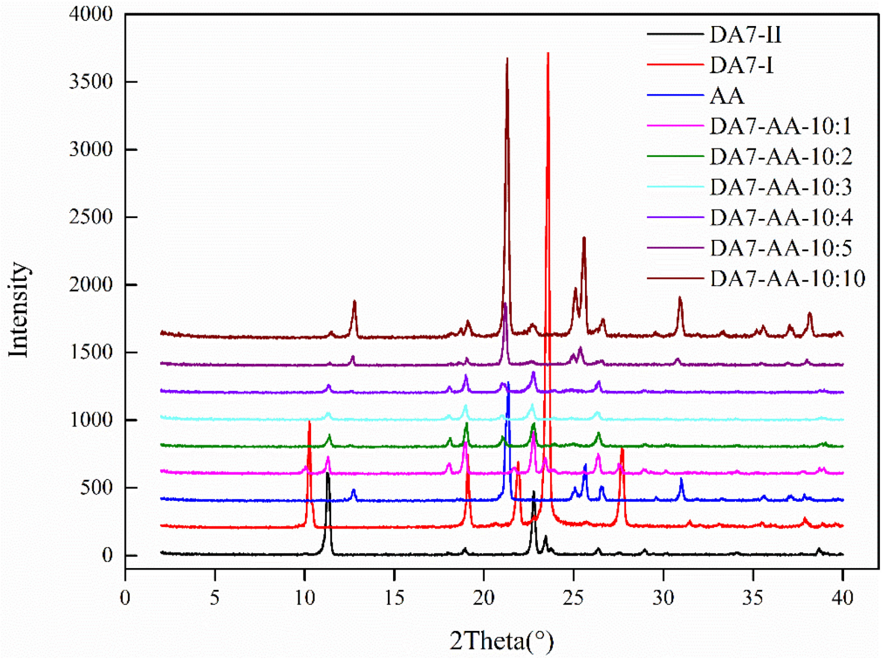

Figure S11 PXRD patterns of DA7 for additive experiments in Table 3(AA as additives)

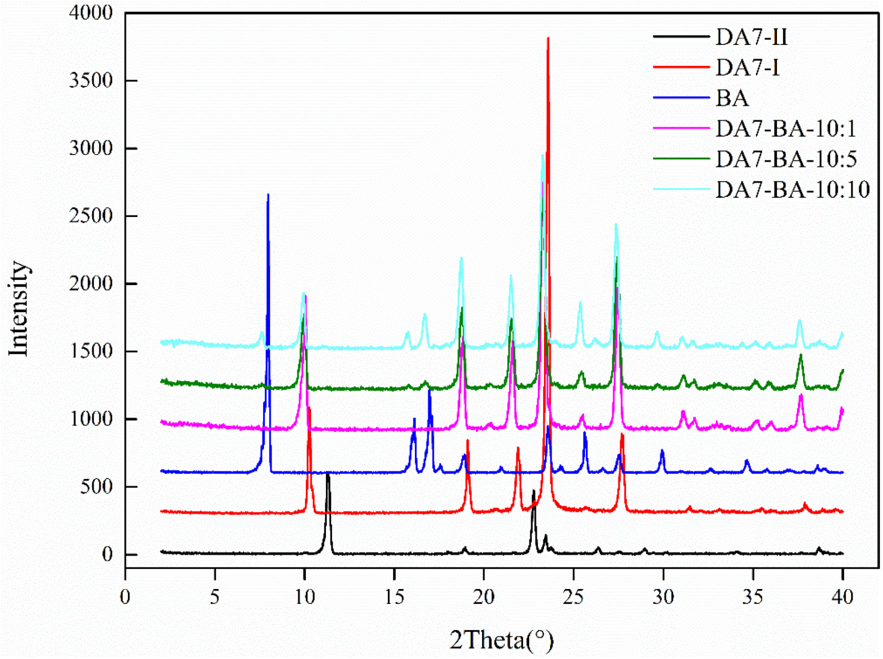

Figure S12 PXRD patterns of DA7 for additive experiments in Table 3(BA as additives)

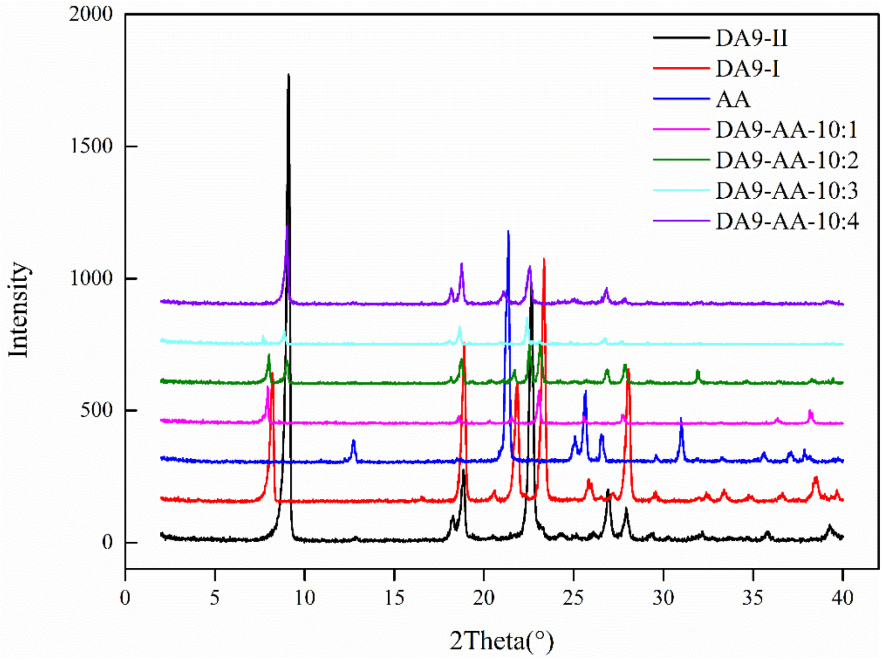

Figure S13 PXRD patterns of DA9 for additive experiments in Table 3(AA as additives)

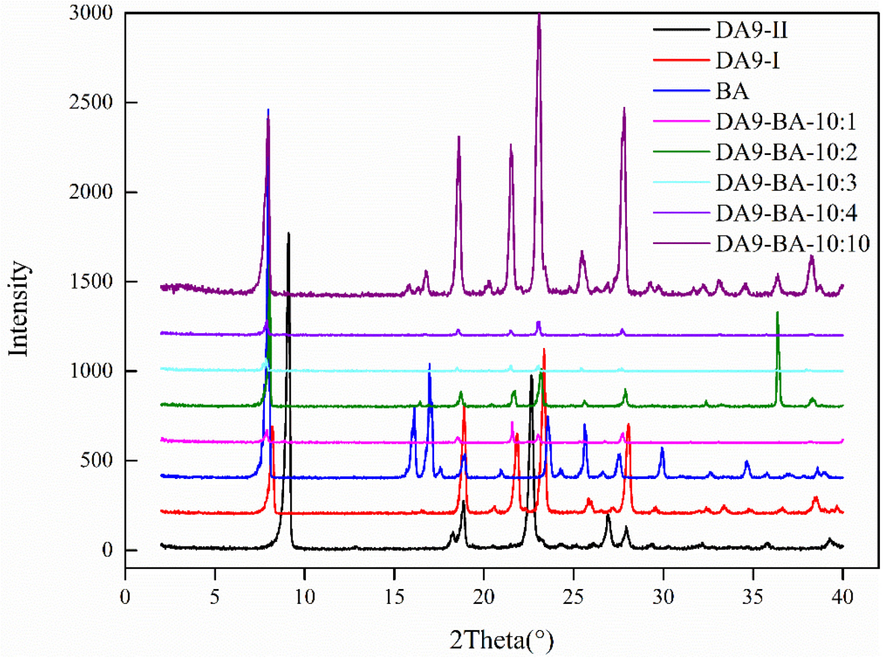

Figure S14 PXRD patterns of DA9 for additive experiments in Table 3(BA as additives)

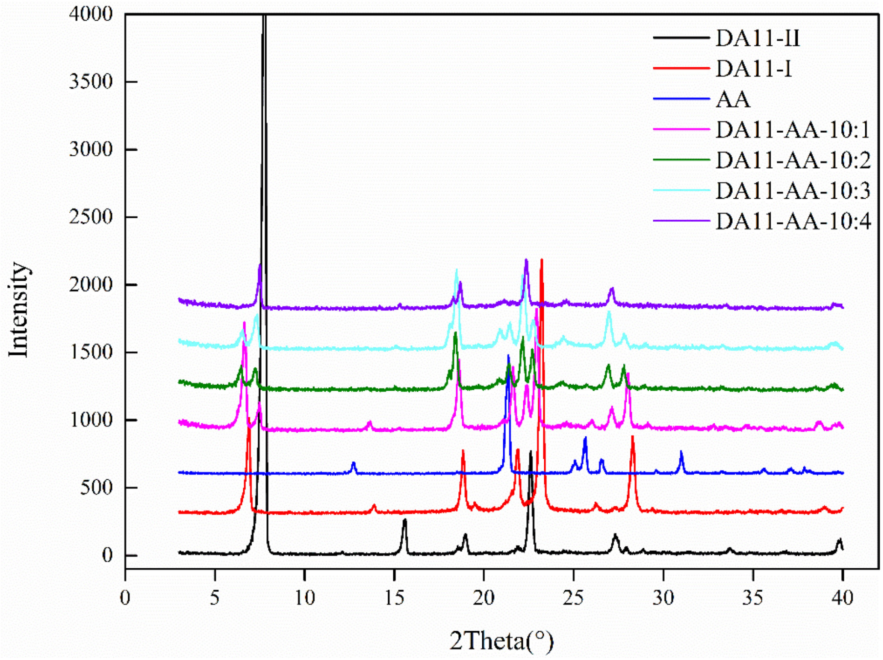

Figure S15 PXRD patterns of DA11 for additive experiments in Table 3(AA as additives)

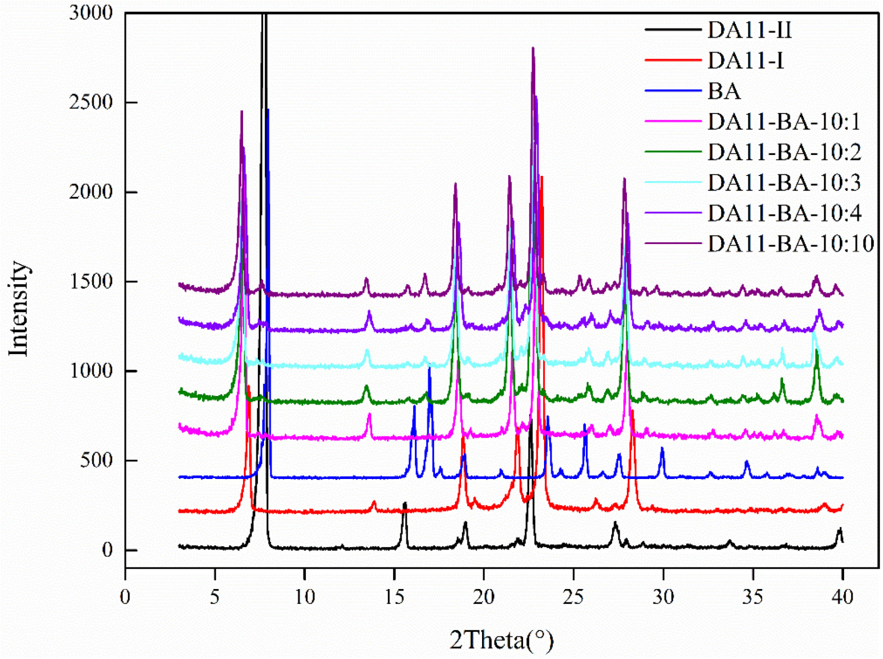

Figure S16 PXRD patterns of DA11 for additive experiments in Table 3(BA as additives)

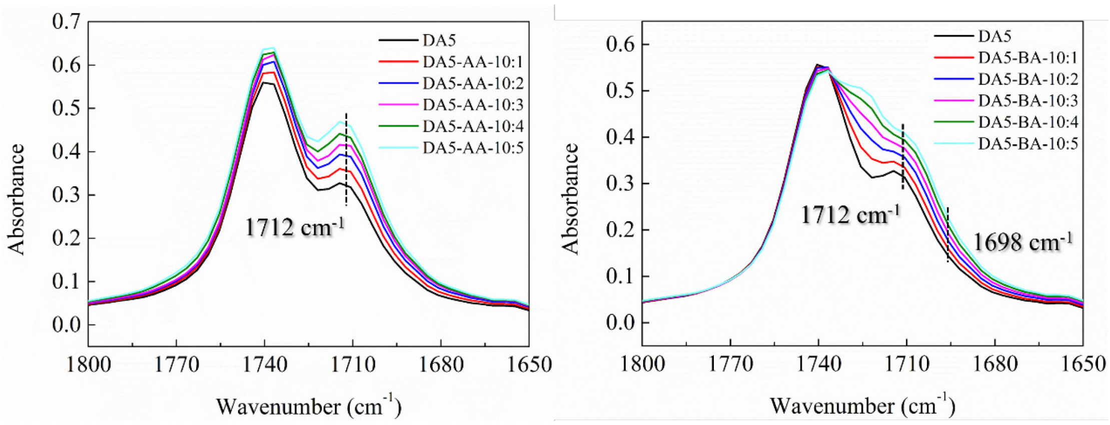

Figure S17 Solution IR spectra of additives in DA5 solution at initial 1.75 M in dioxane over a concentration range. (b) adipic acid; (d) benzoic acid.
